# Sphingosine-1-phosphate receptor 1/5 selective agonist alleviates ocular vascular pathologies

**DOI:** 10.1038/s41598-024-60540-6

**Published:** 2024-04-27

**Authors:** Shinsuke Nakamura, Rie Yamamoto, Takaya Matsuda, Hiroto Yasuda, Anri Nishinaka, Kei Takahashi, Yuki Inoue, Sadao Kuromitsu, Masamitsu Shimazawa, Masahide Goto, Shuh Narumiya, Hideaki Hara

**Affiliations:** 1 Molecular Pharmacology, Department of Biofunctional Evaluation, Gifu Pharmaceutical University, 1-25-4 Daigaku-nishi, Gifu, 501-1196 Japan; 2 Discovery Accelerator, Astellas Pharma Inc., Tsukuba, Japan; 3 Alliance Laboratory for Advanced Medical Research, Kyoto University Graduate School of Medicine, Kyoto, Japan; 4 Pharmaceutical Research and Technology Labs, Astellas Pharma Inc., Yaizu, Japan; 5 Astellas Institute for Regenerative Medicine, Marlborough, MA USA; 6 Department of Drug Discovery Medicine, Kyoto University Graduate School of Medicine, Kyoto, Japan

**Keywords:** Pharmacology, Receptor pharmacology, Angiogenesis

## Abstract

Ocular abnormal angiogenesis and edema are featured in several ocular diseases. S1P signaling via S1P1 likely is part of the negative feedback mechanism necessary to maintain vascular health. In this study, we conducted pharmacological experiments to determine whether ASP4058, a sphingosine 1-phosphate receptor 1/5 (S1P1/5) agonist, is useful in abnormal vascular pathology in the eye. First, human retinal microvascular endothelial cells (HRMECs) were examined using vascular endothelial growth factor (VEGF)-induced cell proliferation and hyperpermeability. ASP4058 showed high affinity and inhibited VEGF-induced proliferation and hyperpermeability of HRMECs. Furthermore, S1P1 expression and localization changes were examined in the murine laser-induced choroidal neovascularization (CNV) model, a mouse model of exudative age-related macular degeneration, and the efficacy of ASP4058 was verified. In the CNV model mice, S1P1 tended to decrease in expression immediately after laser irradiation and colocalized with endothelial cells and Müller glial cells. Oral administration of ASP4058 also suppressed vascular hyperpermeability and CNV, and the effect was comparable to that of the intravitreal administration of aflibercept, an anti-VEGF drug. Next, efficacy was also examined in a retinal vein occlusion (RVO) model in which retinal vascular permeability was increased. ASP4058 dose-dependently suppressed the intraretinal edema. In addition, it suppressed the expansion of the perfusion area observed in the RVO model. ASP4058 also suppressed the production of VEGF in the eye. Collectively, ASP4058 can be a potential therapeutic agent that normalizes abnormal vascular pathology, such as age-related macular degeneration and RVO, through its direct action on endothelial cells.

## Introduction

Chorioretinal vascular diseases share common pathological features based on vascular vulnerability and are a leading cause of blindness due to their potential to cause severe tissue edema, hemorrhage, abnormal angiogenesis, and rhegmatogenous retinal detachment^1,2^. Therapeutic approaches targeting vascular endothelial growth factor (VEGF) have dramatically improved the outcomes of patients with chorioretinal vascular disease^3^. However, all patients do not respond to anti-VEGF therapy, and anti-VEGF therapy may enhance the possibility of ocular and systemic side effects^4,5^. Therefore, novel VEGF-independent factors that can effectively treat abnormal angiogenesis and hyperpermeability need to be identified.

Sphingosine 1-phosphate receptor (S1P), a bioactive sphingolipid, is a critical signaling molecule responsible for regulating various intracellular processes, including cell survival, differentiation, and proliferation^6–8^. S1P exhibits biological effects mainly through five membrane-bound G-protein-coupled receptors, S1P1-S1P5^9^. A recent study that evaluated endothelium-specific S1P1 deficiency showed that the S1P1 signal inhibits angiogenesis in the retina of neonatal mice^10^. S1P signaling via S1P1 likely is part of the negative feedback mechanism necessary to maintain vascular health by resisting VEGF signaling and excessive vascular sprouting^11^. S1P1 appears critical in inhibiting VEGF-dependent angiogenesis and enhancing vascular stability by strengthening the endothelial cell–cell junction.

FTY720, an S1P receptor agonist, has been marketed for the treatment of multiple sclerosis. FTY720 is a non-selective S1P receptor (S1P 1/3/4/5) agonist and may cause S1P3-induced bradycardia and bronchoconstriction^12,13^. An S1P receptor agonist without binding activity to S1P3 could be a therapeutic agent for lesions associated with angiogenesis and increased vascular permeability. We have already succeeded in synthesizing ASP4058, an S1P1/5-selective S1P receptor agonist that exerts no binding activity to S1P3^14^. S1P receptor agonists, which do not show serious side effects, are expected to benefit patients with retinochoroidal vascular disease who do not responsive to anti-VEGF monotherapy when used in combinational therapy with anti-VEGF agents. This study investigates the efficacy of ASP4058 in choroidal neovascularization and retinal vascular hyperpermeability model mice.

## Materials and methods

### Cell culture

We bought primary human retinal microvascular endothelial cells (HRMECs) from Cell Systems (Kirkland, WA, USA). HRMECs were kept in a complete classical medium mixed with CultureBoost-R (Cell Systems) and antibiotics composed of 100 U/mL penicillin (Meiji Seika Pharma Co., Ltd.) and 100 μg/mL streptomycin (Meiji Seika Pharma Co., Ltd., Tokyo, Japan). Before seeding the cells, the culture dishes were coated with attachment factor (Cell Systems). Chinese hamster ovary-K1 (CHO-K1) cells overexpressing human S1P1 receptors (S1P1-CHO) was previously established^14^. S1P1-CHO cells and mock CHO-K1 cells were cultured in Ham’s F12 medium supplemented with 10% FBS, 50 µg/mL streptomycin, 50 U/mL penicillin and 1 mg/mL G418 sulfate. All cells were sustained at 37 °C in a humidified atmosphere with 5% CO_2_.

### Polymerase chain reaction

Conventional polymerase chain reaction (PCR) was utilized to determine the presence of S1P receptors in HRMECs. Total ribonucleic acid (RNA) was extracted from HRMECs using an RNeasy Plus Mini Kit (QIAGEN, Hilden, Germany), and cDNA was synthesized using SuperScript IV VILO Master Mix (Thermo Fisher Scientific, Waltham, MA, USA). Reverse transcription-PCR was then performed with KOD FX (Toyobo, Osaka, Japan), and amplified samples were resolved by agarose gel electrophoresis. The primer sets utilized were as follows: forward, 5′- GCTCTCCGAACGCAACTTC-3′ and reverse, 5′- GCTTCAGGGGTGGTTCGATG-3′ for S1P_1_, forward, 5′- GAGGTCTGAGAATGAGGAATGG-3′ and reverse, 5′- CACTGTCCTGAGGAGCTAGAGG-3′ for S1P_2_, forward, 5′- CGGAGGAGCCCTTTTTCAAC-3′ and reverse, 5′-

TGCCATCACTTGGCATTCAC-3′ for S1P_3_, forward, 5′- ATCATCAGCACCGTCTTCAGC-3′ and reverse, 5′- CTCTACTCCAAGCGCTACATCC-3′ for S1P_4_, forward, 5′- TCACTCGGTTCAAGGCAGCG-3′ and reverse, 5′-GGAGCTTGCCGGTGTAGTTG-3′ for S1P_5_.

### Flow cytometry

Cells were treated with a mouse anti-S1P1 antibody (R&D systems, Minneapolis, MN, USA) for 30 min on ice and then reacted with PE-conjugated anti-mouse immunoglobulin G (IgG; Biolegend, San Diego, CA). As an isotype control, purified mouse IgG2b (Biolegend) was prepared. The cells were analyzed using CantoII and FlowJo software (BD Biosciences, San Jose, CA, USA). Dead cells were stained with 7-amino actinomycin D or SYTOX Blue Dead Cell Stain (Thermo Fisher Scientific) and gated out.

### cAMP assay

HRMECs were cultured and grown at a density of 1 × 10^4^ cells per well in 96-well plates. After overnight serum starvation, the cells were reacted with 10 μM forskolin (Sigma-Aldrich, St. Louis, MO, USA) and 0.5 mM IBMX (Sigma-Aldrich) in the presence of compounds for 10 min at 37 °C and then treated with lysis buffer (50 mM HEPES, 10 mM CaCl_2_, 0.35% Triton X-100). The cAMP concentration in cell lysates was determined by LANCE cAMP 384 kit (PerkinElmer, Shelton, CT, USA) or LANCE Ultra cAMP Kit (PerkinElmer) following the manufacturer’s instructions. The EC_50_ value was calculated via nonlinear regression by GraphPad Prism 8.0.2 (GraphPad Software, San Diego, CA, USA).

### Cell proliferation assay

HRMECs were seeded into a 96-well plate at a density of 2 × 10^3^ cells/well and incubated for 24 h at 37 °C with 5% CO_2_. The medium was replaced with CSC medium containing 10% fetal bovine serum (Biosera, Kansas City, MO, USA) without cell boost and incubated at 37 °C for 24 h. The cells were pre-incubated with ASP4058 for 1 h, and VEGF (10 ng/mL) was added into the medium. Then, HRMECs were incubated for 72 h. After incubation, the cell proliferation rates were analyzed using a cell counting kit (Dojindo, Kumamoto, Japan) per the information from the manufacturer’s protocol. The absorbance at 450/650 nm was recorded using a plate reader (Thermo Fisher Scientific).

### Evaluation of endothelial barrier function

HRMECs were seeded at 1 × 10^5^ cells/well on fibronectin and collagen-coated Transwell inserts (pore size: 0.4 μm, diameter: 6.5 mm) and cultured for two days. The cells were stimulated with VEGF (30 ng/mL), while FITC-conjugated dextran (500 KDa, 1 mg/mL) was added into the insert wells. Medium from the bottom wells was collected over time, and the fluorescence intensity was recorded with a FlexStation 3 microplate reader (Molecular Devices, excitation wavelength 490 nm, emission wavelength 520 nm). To evaluate the effect of ASP4058 on endothelial barrier function, given concentrations of ASP4058 were added 1 h before or 6 h after the addition of VEGF.

### Animals

Eight-week-old male C57BL/6J mice and ddY mice were purchased from Japan SLC (Shizuoka, Japan). They were kept at 24 ± 2 °C under a 12 h light–dark cycle and provided with free access to food and water. All investigations were performed in conformity with the Association for Research in Vision and Ophthalmology statement for the Use of Animals in Ophthalmic and Vision Research. In addition, all animal experiment were approved (approval numbers; 2016–300, 2017–163, 2017–165, 2017–272, 2017–273, 2018–028, and 2018–068) and monitored by the institutional animal care and use committee of Gifu Pharmaceutical University. Ethics statement of animal experimentation; All investigations were performed under protocols approved by the institutional animal care and use committee of Gifu Pharmaceutical University (approval numbers; 2016–300, 2017–163, 2017–165, 2017–272, 2017–273, 2018–028, and 2018–068). In addition, all work was done were conducted according to ARRIVE guidelines (https://arriveguidelines.org).

### Immunostaining

For the in vitro study, HRMECs were seeded at 1 × 10^5^ cells/well on fibronectin- and collagen-coated Transwell inserts and cultured for 2 days. The cells were stimulated with VEGF (30 ng/mL) for 3 h after 1 h pretreatment with ASP4058 (10 nM). To examine the expression of S1P1 receptor, HRMECs were seeded at 1 × 10^5^ cells/well on chamber slide and cultured for one day. Then, the cells were fixed with 4% paraformaldehyde (PFA; pH 7.4) for 10 min at room temperature (RT) and treated with 0.1% TritonX-100 for 10 min at RT. The cells were blocked in 5% goat serum for 1 h and incubated overnight at 4 °C with a mouse monoclonal antibody against VE-cadherin (1:100, Merck, Germany) or S1P1/EDG rabbit polyclonal antibody (1:100, Santa cruz biotechnology, TX, USA). After that, the cells were incubated with Alexa594-goat anti-mouse IgG (1:1000, Thermo Fisher Scientific) or Alexa594-goat anti-rabbit IgG (1:1000, Thermo Fisher Scientific) for 1 h and mounted with medium containing DAPI (Thermo Fisher Scientific). The stained cells were photographed on an Axio Imager microscope (Carl Zeiss Microscopy GmBH, Germany) or a confocal fluorescence microscope system (LSM-710, Carl Zeiss Microscopy GmBH), and maximum intensity projection was performed on images from confocal microscopy.

For the in vivo study, the eyes were removed and fixed with 4% PFA for 24 h at 4 °C and then dehydrated in 25% sucrose at 4 °C for two days. The eyes were then embedded in optimal cutting temperature compound (Sakura Finetek Japan) and instantly frozen with liquid nitrogen. Then, 10 µm thick chorioretinal sections were cut using a cryostat (Leica Microsystems, Bensheim, Germany) and put on the glass slides (MAS COAT; Matsunami Glass, Osaka, Japan).

The sections were blocked in goat serum (Vector Laboratories, Burlington, USA) and then reacted with S1P1/EDG rabbit polyclonal antibody (1:200, Abcam, Cambridge, MA, USA), fluorescein-labeled griffonia simplicifolia lectin I (GSL I) isolectin B4 (IB4) (20 µg/mL: Vector Laboratories), and glutamine synthetase (GS) mouse polyclonal antibody (1:100, Merck Millipore, Burlington, USA) Alexa Flour-647 rat monoclonal CD31 antibody (1:50, Biolegend, San Diego, CA, USA) overnight at 4 ℃. The next morning, the samples were rinsed with phosphate-buffered saline (PBS) and covered with Alexa Fluor 488 goat anti-mouse IgG (1:1000, Thermo Fisher Scientific) and Alexa Fluora-546 Goat anti-rabbit IgG (1:1000, Thermo Fisher Scientific) for 1 h at RT. The nuclei were counterstained with Hoechst 33,342 (1:1000; Thermo Fisher Scientific) for 15 min at RT. After rinsing with PBS, the slides were mounted with Fluoromount. The immunofluorescence images were obtained with a confocal microscope (FLUOVIEW FV10i; Olympus, Tokyo, Japan).

### Laser-induced choroidal neovascularization (CNV) model

Eight-week-old male C57BL/6J mice were anesthetized by using a mixture of xylazine (2.5 mg/kg; Bayer Healthcare, Tokyo, Japan) and ketamine (43.8 mg/kg; Daiichi Sankyo Propharma, Tokyo, Japan) by intramuscular injection. The pupils were dilated with 0.5% phenylephrine and 0.5% tropicamide (Santen Pharmaceuticals Co., Ltd., Osaka, Japan). After dilation, laser photocoagulation was performed using a 647 nm laser (MC500, NIDEC, Kyoto, Japan). Laser spots were generated using a red laser (647 nm wavelength, 50 µm spot size, 100 mW power and 100 ms duration) on day 0. Six laser spots were applied to around the optic nerve. Successful laser photocoagulation was verified by the presence of bubble formation, indicating the rupture of Bruch’s membrane.

### Quantitative real-time polymerase chain reaction (qRT-PCR)

The total RNA in the retinal pigment epithelium (RPE)-choroid complex from mice was isolated using the NucleoSpin RNA kit (Takara, Shiga, Japan) per the information from the manufacturer’s protocol. Also, the total RNA from ARPE-19 cells was performed in the same manner. The RNA concentration was measured by using NanoVue Plus (GE HealthCare Japan, Tokyo, Japan), and cDNA was synthesized by PrimeScript RT Reagent kit (Takara). To determine the *S1p1 and VEGF* mRNA expression, SYBR Premix Ex TaqII (Takara) and TP8000 Thermal Cycler Dice Real-Time system (Takara) were used. For housekeeping, the expression of *β-actin* was used.

The PCR primer sequences for murine tissues used were: *S1p1* forward, 5′-CACCGGCCCATGTACTATTT-3′ and reverse, 5′-GACTGCCCTTGGAGATGTTC-3′ and *β-actin* forward: 5′-TCAAGATCATTGCTCCTCCTG -3′, reverse: 5′- CTGCTTGCTGATCCACATCTG -3′.

The PCR primer sequences for human cells used were: *Vegf* forward, 5′-TCTACCTCCACCATGCCAAGT-3′ and reverse, 5′-GATGATTCTGCCCTCCTCCTT -3′ and *β-actin* forward: 5′-TCAAGATCATTGCTCCTCCTG-3′, reverse: 5′-CTGCTTGCTGATCCACATCTG-3′.

### Immunoblotting

Western blotting was performed as previously described^15^. To evaluate the effect of ASP4058 on HRMECs in detail, HRMECs were seeded into a 24-well plate at a density of 2 × 10^4^ cells/well and cultured for 24 h. Then, we changed the medium to 1% FBS containing CSC medium and incubated for 6 h. HRMECs were pre-incubated with ASP4058 for 1 h, and treated with VEGF (10 ng/mL). After incubating for 5 min, HRMECs were treated with cell lysis buffer. The eyes were removed, and RPE-choroid complexes were rapidly frozen with liquid nitrogen. The tissues were put into cell lysis buffer and homogenized using a homogenizer (Physcotron; Microtec Co. Ltd., Chiba, Japan). The cell lysis buffer comprised a radioimmunoprecipitation buffer containing 150 mM sodium chloride (Kishida Chemical, Osaka, Japan), 1% Igepal CA-630 (Sigma-Aldrich), 0.5% sodium deoxycholate (Wako, Osaka, Japan), 0.1% sodium dodecyl sulfate (SDS) (Wako), 50 mM Tris-hydrochloride (Nacalai Tesque, Kyoto, Japan), phosphatase inhibitor cocktails (Sigma-Aldrich), and protease inhibitor cocktail (Sigma-Aldrich). The homogenized and collected samples were centrifuged at 12,000×*g* for 20 min at 4 °C. After configuration, their supernatant was collected, and the protein concentrations were measured using the bicinchoninic acid protein assay kit (Pierce Biotechnology, Rockford, IL, USA) with bovine serum albumin as the standard, following the manufacturer’s protocol. The sample buffer (Wako) and protein samples were admixed at a ratio of 1:3, and the mixtures were boiled for 5 min. The samples were separated by a gradient of 5–20% SDS-PAGE (Wako) and transferred to polyvinylidene difluoride membranes (Millipore, Bedford, MA, USA). After the membranes were rinsed with tris-buffered saline containing 0.05% Tween20 (Bio-Rad, Hercules, CA, USA), they were treated with Blocking One-P (Nacalai Tesque) for 30 min at RT. After blocking, the membranes were incubated overnight at 4 °C with primary antibodies. The primary antibodies used were: phosphor-p38 mitogen-activated protein kinase (p38) rabbit polyclonal antibody (1:1000, Cell Signaling Technology, Danvers, MA, USA), p38 rabbit polyclonal antibody (1:1000, Cell Signaling Technology), phospho-src tyrosine kinase (src) rabbit polyclonal antibody (1:1000, Cell Signaling Technology), src rabbit polyclonal antibody (1:1000, Cell Signaling Technology) S1P1/EDG rabbit polyclonal antibody (1:1000, Abcam), VEGF rabbit polyclonal antibody (1:1000, Merck Millipore) and β-actin mouse monoclonal antibody (1:2000, Sigma-Aldrich).

The membranes were then washed and incubated with horseradish peroxidase (HRP)-conjugated goat anti-mouse IgG (1:2000, Thermo Fisher Scientific) and HRP- conjugated goat anti-rabbit IgG (1:2000, Thermo Fisher Scientific). The immunoreactive bands were made visible using ImmunoStar LD (Wako). The band intensity was analyzed using LAS-4000 Luminescent Image Analyzer (Fuji Film Co. Ltd., Tokyo, Japan).

### Drug administration

ASP4058 was obtained from Astellas Pharma Inc. (Tokyo, Japan). In the in vivo study, ASP4058 was suspended in 0.5% sodium carboxymethyl cellulose (Wako) and orally administered in all experiments. In the choroidal neovascularization (CNV) model, 0.03 or 0.3 mg/kg ASP4058 was orally dosed daily before laser irradiation (Figs. 4 and 6). Aflibercept was purchased from Bayer (Leverkusen, Germany) and intravitreally injected at 20 µg/eye immediately after laser irradiation. PBS was injected in the control group (Fig. 4D–F). In the retinal vein occlusion (RVO) model, ASP4058-treated mice were divided into three groups. The mice in the first group were administered ASP4058 (0.003, 0.01, 0.03, 0.3, 1, and 3 mg/kg) just after laser irradiation (Fig. 5A–D). The mice in the second group were administered 0.3 mg/kg ASP4058 twice (12 h before and just after laser irradiation; Fig. 5E–F). Furthermore, 0.3 mg/kg ASP4058 was administered twice seven days after laser irradiation (Fig. 5E,G).

### Fluorescein angiography

Fourteen days after the CNV induction, the mice were anesthetized with a mixture of xylazine and ketamine with an intramuscular injection. The pupils were dilated using 0.5% phenylephrine and 0.5% tropicamide, and 0.1% sodium hyaluronate (Santen Pharmaceuticals Co., Ltd.) was applied to prevent desiccation. After the tail vein was injected with saline to confirm successful injection, 0.1 mL fluorescein (10 mg/mL; Alcon Japan Ltd., Tokyo, Japan) was injected, and its leakage was confirmed using MicronIV Retinal Imaging Microscope (Phoenix Research Laboratories, Pleasanton, CA). Fluorescent fundus photographs were obtained at one and three minutes after the fluorescein administration, and the images were defined as early or late phase. The following scores were used as previously described^16^.

### Measurement of CNV area

After performing fluorescein angiography, the mice were subsequently perfused with 0.5 mL PBS solution containing FITC-conjugated dextran (MW = 2000 kDa, 20 mg/mL). After perfusion, the eyes were removed and immediately fixed with 4% PFA for 12 h at 4 °C. The RPE-choroid complex was flat-mounted, and these flat-mounted preparations were mounted on slide glass (Matsunami Glass) using Fluoromount (Diagnostic BioSystems, Pleasanton, CA, USA). The CNV regions were photograghed using a confocal microscope. The CNV areas were measured in a blind manner.

### RVO model

The RVO mice were created using ddY mice as described in detail^17^ The mice were anesthetized with a mixture of xylazine (6 mg/kg) and ketamine (120 mg/kg). Three retinal veins were photocoagulated to induce retinal edema using the MicronIV imaging guide laser. Mice were injected with rose bengal (8 mg/mL; Wako) through the tail vein, and ten to 15 laser irradiations (532 nm wavelength, 50 µm spot size, 5 s duration, and 50 mW power) were delivered to the veins at three disk diameters from the optic disk. Successful occlusions were confirmed by fundus imaging one day after the laser occlusion.

### Histological analysis

Histological analyses of the RVO model were performed using retinal cross-sections stained with hematoxylin and eosin (H&E), as previously described^17^. One day after vein occlusion, the RVO model mice were euthanized by cervical dislocation, and the eyes were removed and fixed in 4% PFA for 48 h at 4 °C. Following embedding in paraffin, a 5 µm thick section was sliced through the optic disk and subjected to H&E. The stained sections were photographed using a fluorescent microscope (BZ-X710; Keyence, Osaka, Japan). The thickness of the inner nuclear layer (INL) at 240–1920 nm from the optic nerve head was measured using ImageJ (National Institutes of Health, Bethesda, MD, USA).

### Evaluation of retinal non-perfusion areas

One, seven, eight, or 14 days after RVO, the mice were subsequently perfused with 0.5 mL PBS solution containing FITC-conjugated dextran (MW = 2000 kDa, 20 mg/mL), and the eyes were enucleated. The retinas were isolated from the eyes and fixed with 4% PFA for 7 h. After fixing, the samples were mounted with Fluoromount and visualized utilizing Metamorph (Universal Imaging Corp., Downingtown, PA, USA). The retinal non-perfused regions were measured using ImageJ.

### Cell culture

Human-derived retinal pigment epithelium cell line (ARPE-19) was obtained from American Type Culture Collection (Manassas, VA, USA) and cultured in Dulbecco’s modified Eagle’s medium (DMEM)/F-12 (FUJIFILM Wako Pure Chemical Corporation, Osaka, Japan) containing 10% FBS, 100 U/mL penicillin and 100 µg/mL streptomycin. Cells were grown and maintained in a humidified atmosphere of 95% air and 5% CO_2_ at 37%. The cells were passaged by trypsinization every 4 days.

### Hypoxic exposure to ARPE-19

ARPE-19 were seeded at a density of 7.5 × 10^4^ cells per well in 24-well plates and incubated for 24 h at 37 °C with 5% CO_2_. The medium was replaced with DMEM/F12 without FBS, and the cells were treated with ASP4058 for 1 h. For hypoxic exposure, the cells were incubated in an oxygen-free incubator (94% N_2_, 1% O_2_, 5% CO_2_) for 6 h.

### Statistical analyses

Data are expressed as the mean ± standard error of the mean (SEM). In cases the data were not normally distributed, non-parametric tests were performed. Levene’s tests were used for equal variances. Statistical analyses were performed using SPSS version 15.0 for Windows (Japan Inc., Tokyo, Japan) or GraphPad Prism 8.0.2 or 10.1.2 (GraphPad Software). Comparisons between group means were conducted utilizing the Student’s* t*-test and for multiple comparisons, data were analyzed with one-way ANOVA followed by Dunnett’s test for data with equal variance and Kruskal–Wallis test with the Bonferroni correction. *P* < 0.05 was considered statistically significant.

## Results

### ASP4058 functions as an S1P1 agonist in HRMECs

**Figure 1:**
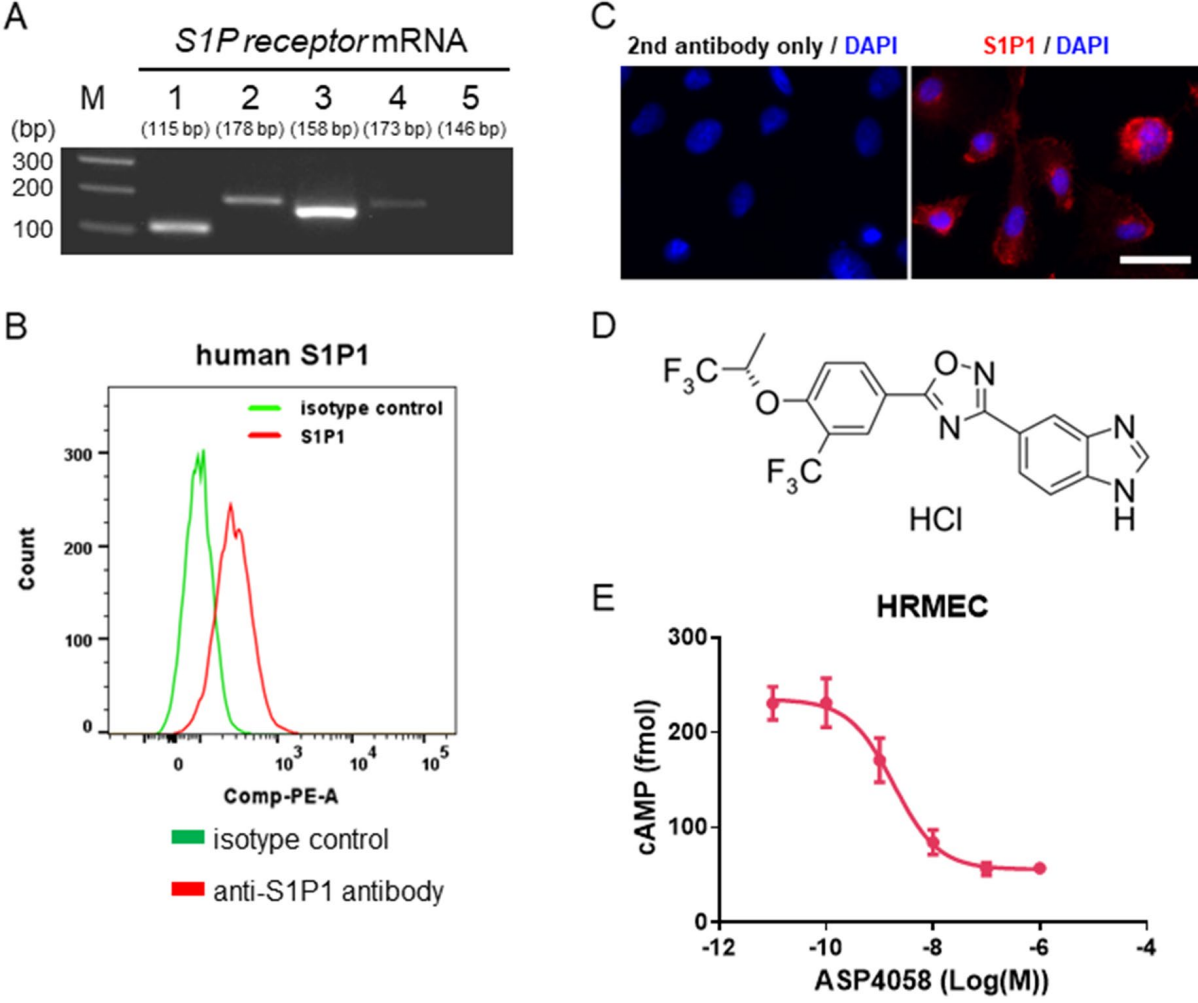
ASP4058 functions as an S1P1 agonist in HRMEC. ASP4058, an S1P1 and S1P5 selective agonist, exerts agonistic effects on HRMEC via S1P1. (**A**) RT-PCR analysis showing all S1P receptor subtypes mRNA expression except for S1P5 in HRMECs. (**B**) Flow cytometry analysis showing the S1P1 cell surface expression in HRMECs. Data are shown as a histogram overlay: isotype control-stained cells (green) and anti-S1P1 antibody-stained cells (red). (**C**) The representative immunofluorescence image of S1P1 receptor (red) and DAPI (blue) of HRMECs. Scale bar = 30 µm. (**D**) Chemical structure of ASP4058. (**E**) ASP4058 inhibited forskolin-induced cAMP accumulation in HRMECs. Data are presented as the mean ± SEM (n = 5).

To investigate whether S1P1 could be a potential drug target for retinochoroidal vascular disease, we investigated the pharmacological effects of the S1P1 agonist ASP4058 in the primary cultures of HRMECs. First, we investigated the S1P receptors expression in HRMECs and detected all S1P receptor subtypes mRNA expression other than S1P5 and the cell surface expression of S1P1 (Fig. 1A–C). Supplementary Fig. 1 indicate that the experimental system used in this study guarantees the specificity of S1P1. The chemical structure of ASP4058 is shown in Fig. 1D. We hypothesized that ASP4058 could act selectively on S1P1 in HRMECs. We evaluated the S1P1 agonistic activity of ASP4058 on HRMECs by assessing its effect on forskolin-induced cAMP production. ASP4058 inhibited cAMP production concentration-dependently, with an EC_50_ value of 1.8 nM (Fig. 1E). To confirm whether this effect was mediated via S1P, we evaluated the effect of FTY720 phosphate (FTY720-P), an S1P analog, in the same assay system and found that FTY720-P also suppresses cAMP production. In addition, the effects of ASP4058 and FTY720-P were both blocked by simultaneous treatment with TASP0277308, an S1P1 antagonist, suggesting that these compounds suppress cAMP production through their agonistic effects on S1P1 (Supplementary Fig. 2). Taken together, these results suggest that ASP4058 function as an S1P1 agonist in HRMECs.

### ASP4058 attenuated VEGF-induced HRMEC proliferation and hyperpermeability

**Figure 2:**
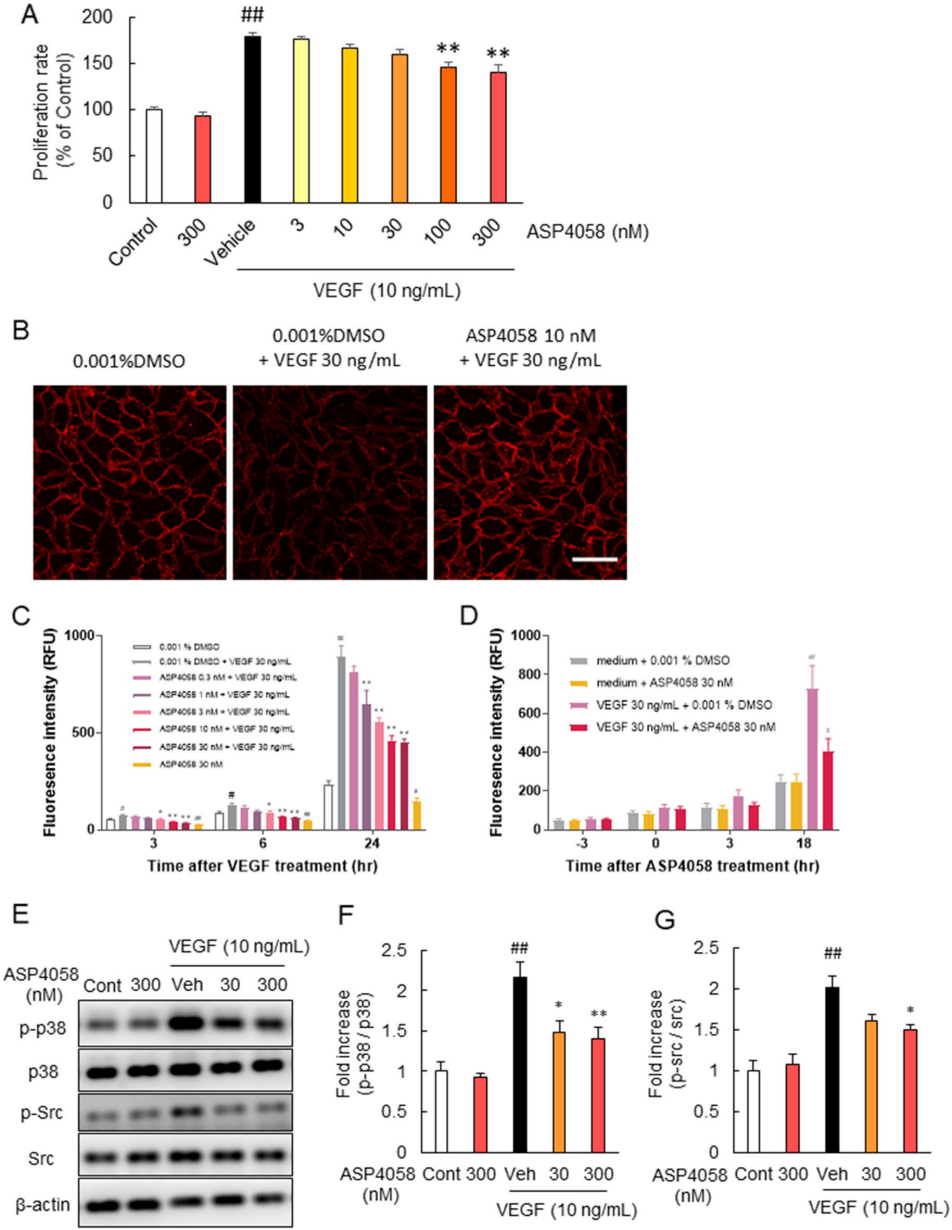
ASP4058 attenuated HRMEC proliferation and hyperpermeability induced by VEGF. ASP4058 reduced HRMECs proliferation and hyperpermeability induced by VEGF. (**A**) ASP4058 decreased VEGF-induced HRMECs proliferation concentration-dependently. Data are shown as the mean ± SEM (n = 6). ^**^; *p* < 0.01 vs. vehicle-treated group, ^##^; *p* < 0.01 vs. control group (Tukey’s test). (**B**) Representative immunofluorescence micrographs analyzing VE-cadherin expression and localization in the VEGF-stimulated HRMEC monolayer. HRMEC monolayers were left untreated (left panel) or treated with 30 ng/mL VEGF (middle and right panel) after pretreatment with 0.001% DMSO (left and middle panel) or 10 nM ASP4058 (right panel) and stained with antibody against VE-cadherin. Scale bar = 50 µm. (**C**, **D**) Pre- or post-treatment with ASP4058 inhibited VEGF-induced hyperpermeability. HRMEC monolayer cultured on Transwell inserts were permeabilized by treatment with 30 ng/mL VEGF, and measured for permeability by diffusing FITC-conjugated dextran (500 KDa) across the insert. (**C**) Treatment with ASP4058 1 h before VEGF treatment inhibited the increase of permeability induced by VEGF. RFU, relative fluorescent units. Data are shown as the mean ± SEM (n = 5). ^*^; *p* < 0.05, ^**^; *p* < 0.01 vs. 0.001% DMSO + 30 ng/mL VEGF group (Dunnett’s test), ^#^; *p* < 0.05, ^##^; *p* < 0.01 vs. 0.001% DMSO group (Student’s *t*-test). (**D**) ASP4058 post-treatment from 3 h after VEGF treatment suppressed VEGF-induced hyperpermeability. Data are presented as the mean ± SEM (n = 6). ^#^; *p* < 0.05, ^##^; *p* < 0.01 vs. 30 ng/mL VEGF + 0.001% DMSO group (Student’s *t*-test). (**E**–**G**) ASP4058 treatment 1 h before VEGF treatment inhibited VEGF-induced phosphorylation of p38 (**F**) and Src (**G**). Data are shown as the mean ± SEM (n = 6). ^##^; *p* < 0.01 vs. Vehicle-treated group, ^*^; *p* < 0.05, ^**^; *p* < 0.01 vs. Control group (Tukey’s test).

Next, we investigated the cell proliferation rate of HRMECs using WST assay. The exposure to VEGF significantly increased the proliferation of HRMECs. ASP4058 inhibited VEGF-induced cell proliferation compared to VEGF alone in a concentration-dependent manner (Fig. 2A). We examined whether ASP4058 could suppress VEGF-induced vascular hyperpermeability. To estimate the effect of ASP4058 on the endothelial cell–cell junctions, we examined the expression and localization of VE-cadherin in HRMEC monolayers by immunofluorescent staining. As previously reported^13^, we observed a decrease in VE-cadherin expression at cell junctions after VEGF stimulation. This VEGF-induced reduction in junctional VE-cadherin staining was blocked by pretreatment with ASP4058 (Fig. 2B). We examined endothelial permeability using the Transwell permeability assay. HRMECs were monolayered on the Transwell inserts, and their permeability was measured by diffusion of FITC-conjugated dextran (500 KDa) across the insert. Pretreatment with ASP4058 inhibited this VEGF-induced hyperpermeability concentration-dependently (Fig. 2C). To examine whether ASP4058 has a therapeutic effect on VEGF-induced hyperpermeability, ASP4058 was added 3 h after VEGF treatment. We found that ASP4058 significantly inhibited hyperpermeability even when treated after VEGF stimulation (Fig. 2D). Furthermore, we investigated the effect of ASP4058 on the activation of VEGF-VEGFR2 pathway using Western-blot analysis. The treatment of ASP4058 inhibited the phosphorylation of p38 and Src which mean the hyperactivation of endothelial cells (Fig. 2E–G).

### Expression of S1P1 in the CNV model mice

**Figure 3:**
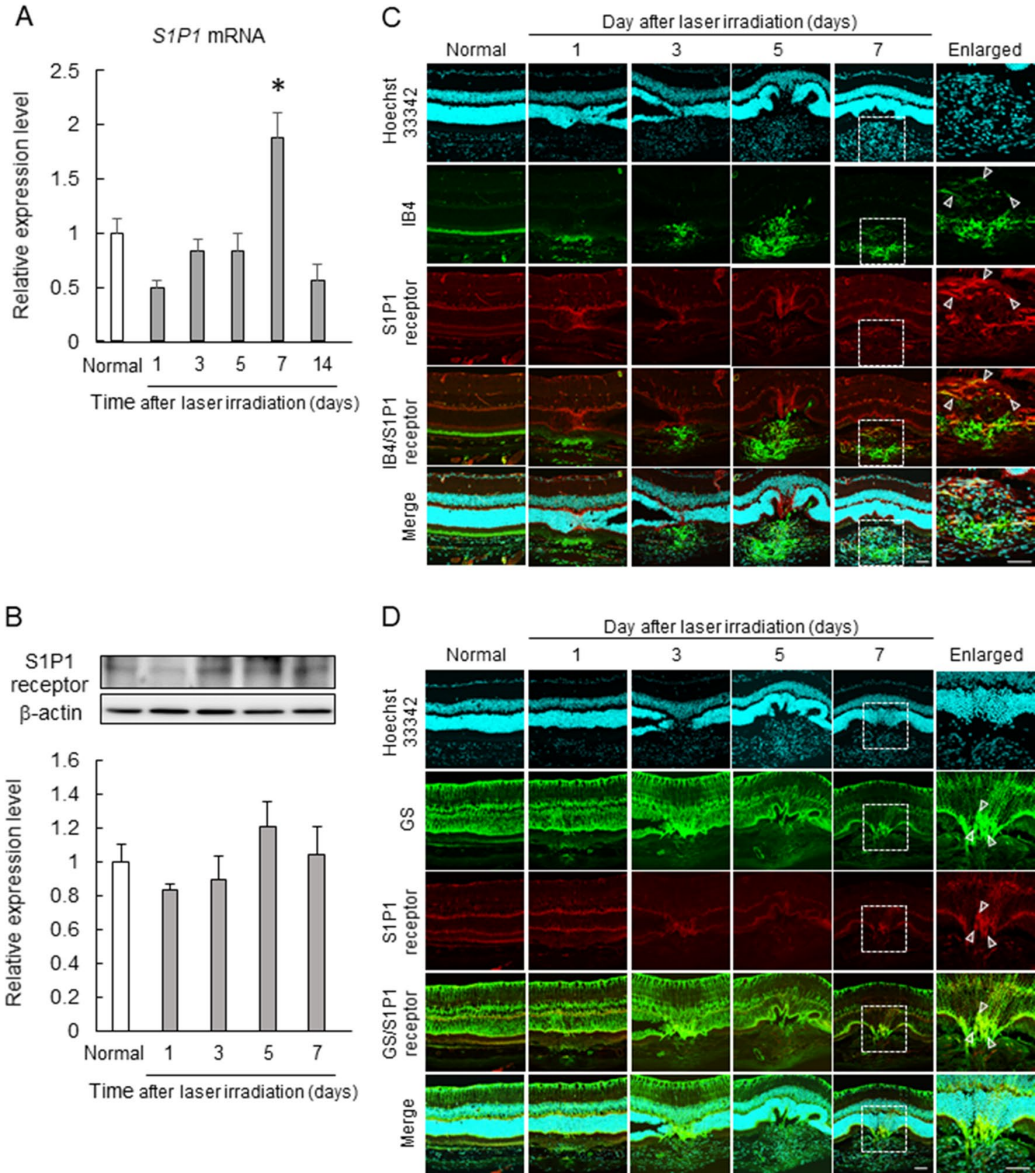
Expression and localization of S1P1 in the laser-induced CNV model. CNV induction affected the expression level and localization of S1P1. The expression level of *S1p1* mRNA at one, three, five, seven, and 14 days after laser irradiation (**A**) and S1P1 protein at one, three, five, and seven days after laser irradiation (**B**) in the RPE-choroid complex. Data are presented as the mean ± SEM (n = 5–10). *; *p* < 0.05 vs. normal group (Games–Howell post-hoc test). (**C**, **D**) Immunohistochemistry of Hoechst 33,342 (cyan), S1P1 (red), IB4 (green) (**C**), and GS (green) (**D**); insets are enlarged image of the enclosed area. Scale bars show 50 µm.

To elucidate the role of S1P1 in chorioretinal pathologies, we examined the changes in the mRNA and protein expression levels of S1p1 on a laser-induced CNV model, a common model of exudative AMD. In the RPE-choroid complex, the mRNA of *S1p1* tended to decrease with a temporary increase only five days after laser irradiation (Fig. 3A), and S1P1 protein was slightly kept at low levels from one to seven days after laser irradiation (Fig. 3B). Next, we investigated the localization of S1P1 in the CNV model using dual immunofluorescence staining of S1P1 with IB4, a marker of endothelial cells, or GS, a marker of Müller cells. From 1 day after laser irradiation, S1P1 was localized with expression of IB4 (Fig. 3C); as with IB4, S1P1 was expressed on CD31 positive cells, supporting the results in Fig. 3C (Supplementary Fig. 3). In addition, S1P1 also co-localized with GS, a marker of Müller cells from 1 day after laser irradiation (Fig. 3D). As a result, the expression of S1P1 co-localized with the expression of IB4 and GS at the CNV lesion (Fig. 3C–D).

### ASP4058 suppressed vascular leakage and CNV formation in the CNV model mice

**Figure 4:**
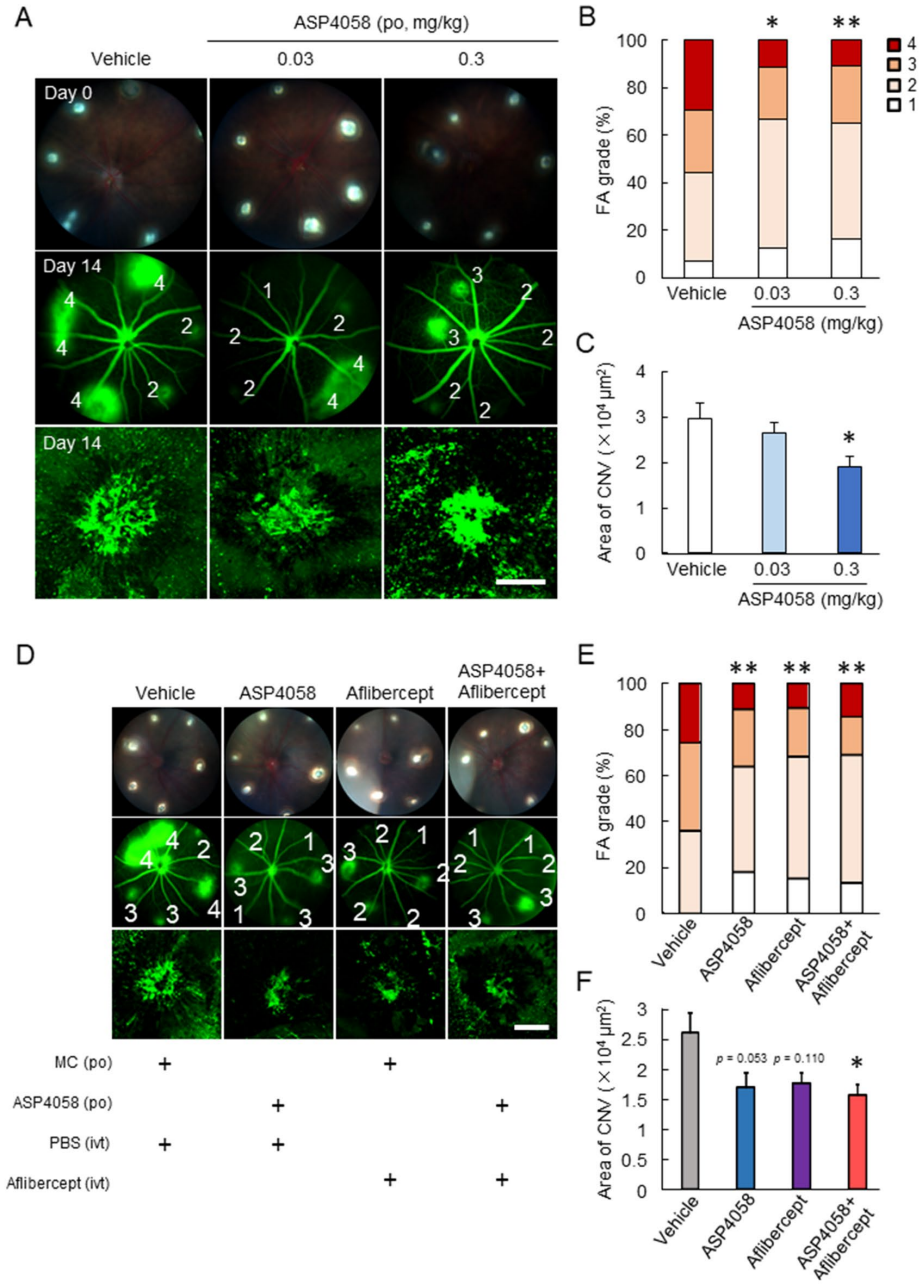
Anti-angiogenic effects of ASP4058 on the CNV model mice. ASP4058 showed anti-angiogenic effects on CNV development in the laser-induced CNV model. (**A**–**C**) The laser-induced CNV model mice were orally administered ASP4058 (0.03 or 0.3 mg/kg) once a day. (**A**) Representative fundus images immediately after laser irradiation (day zero) and after fluorescein injection (day 14) with grades (1–4) and CNV lesion visualized by FITC-dextran. (**B**) Quantitative data of vascular leakages (grade 1–4) of each spot. **; *p* < 0.01 vs. vehicle-administered group (Kruskal–Wallis test). (**C**) Quantification of the mean size of CNVs. The scale bar indicates 100 µm. Data are presented as the mean ± SEM (n = 11 or 12). **; *p* < 0.01 vs. vehicle-administered group (Dunnett’s test). (**D**–**F**) Murine laser-induced CNV model were orally administered 0.3 mg/kg ASP4058 once a day and/or intravitreal administered anti-VEGF agent, aflibercept. (**D**) Representative fundus images immediately after laser irradiation (day zero) and after fluorescein injection (day 14) with grades and the CNV lesion. (**E**) Quantitative data of vascular leakages (grade 1–4) of each spot. **; *p* < 0.01 vs. vehicle-administered group (Kruskal–Wallis test). (F) Quantification of the mean size of CNVs. The scale bar shows 100 µm. Data are presented as the mean ± SEM (n = 11–14). *; *p* < 0.05 vs. vehicle-administered group (Tukey’s test).

To evaluate the effectiveness of ASP4058 on CNV development, ASP4058 (0.03 and 0.3 mg/kg) was orally administered in the CNV model. The oral administration of 0.3 mg/kg ASP4058 significantly reduced the fluorescein leakage from CNV and CNV formation (Fig. 4A–C). We compared the anti-angiogenic effect of ASP4058 to that of aflibercept, an anti-VEGF drug. The oral administration of 0.3 mg/kg ASP4058 and the single intravitreal administration of aflibercept suppressed vascular leakage and tended to decrease CNV formation (Fig. 4D–F). The combination administration of these agents significantly reduced CNV development and vascular leakage. The combination-therapy group showed a trend toward increased efficacy relative to the monotherapy group, but this was not significant statistically.

### ASP4058 reduced the formation of retinal edema and non-perfused region in the RVO model mice

**Figure 5:**
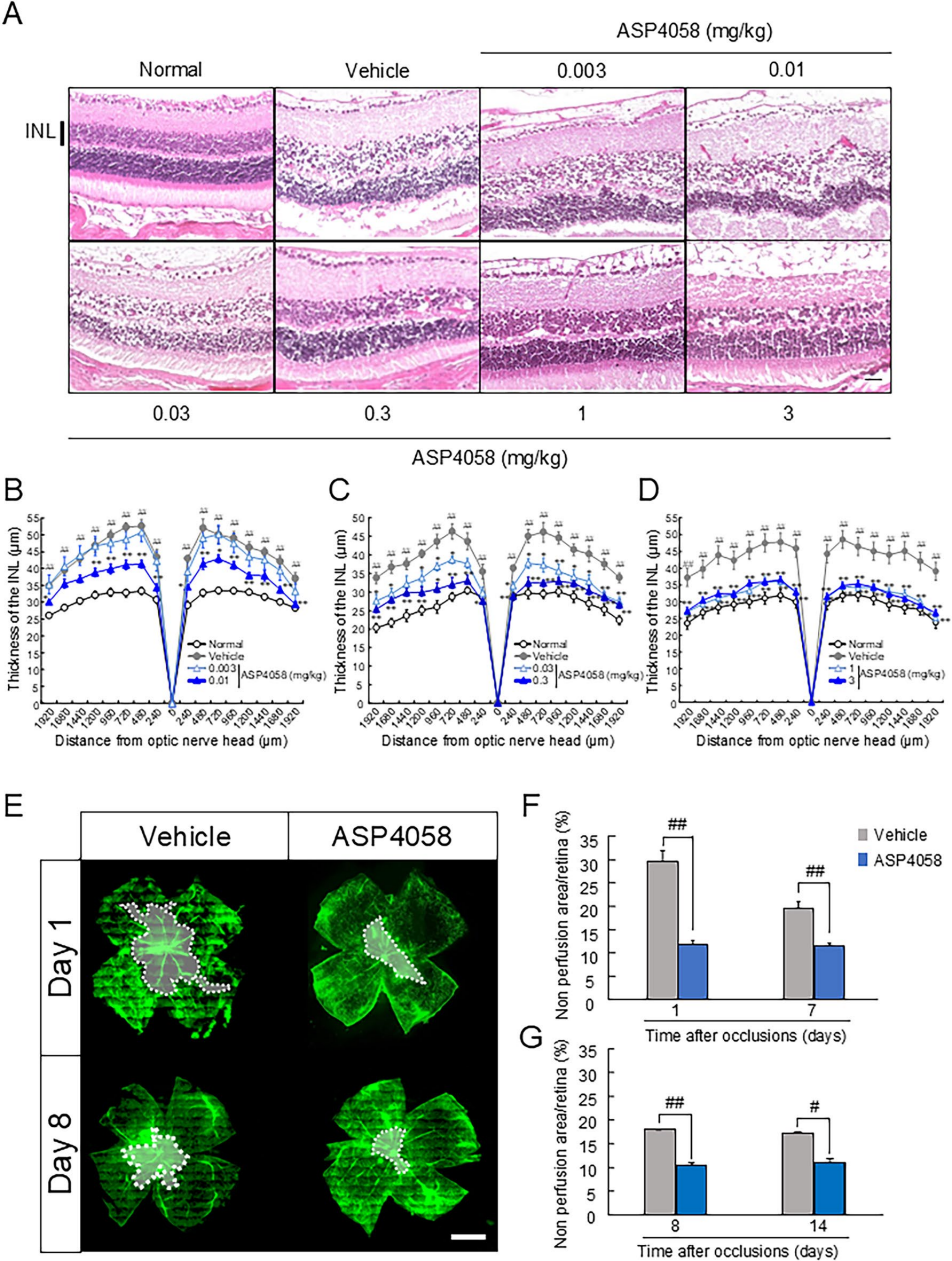
Effect of ASP4058 on the formation of retina edema and non-perfused region in the RVO model mice. ASP4058 suppressed the formation of retinal edema and non-perfusion area in the RVO model. (**A**) Photomicrographs of representative H&E-stained retinal sections of normal, vehicle, and ASP4058 groups at 0.003, 0.01, 0.03, 0.3, 1, and 3 mg/kg orally administered just after laser irradiation. Images were taken at 500 µm from the optic nerve head. Scale bar indicates 50 µm. (**B**–**D**) Quantitative data of the thickness of the INL. Each graph contains 0.003 and 0.01 (**B**), 0.03 and 0.3 (**C**), and 1 and 3 mg/kg (**D**) ASP4058. Data are presented as the mean ± SEM (n = 10). ^*^; *p* < 0.05, ^**^; *p* < 0.01 vs. vehicle-treated group (Dunnett’s T3 test), ^##^; *p* < 0.01 vs. normal group (Welch’s *t*-test). (**E**–**G**) The effect of ASP4058 at 0.3 mg/kg on the formation of a non-perfusion area. ASP4058 was orally administered 12 h before and just after laser irradiation (early phase) or twice seven days after occlusion (late phase). To examine the effect of ASP4058 treatment in the early and late phase, the retinas were collected at days one or seven, and eight or 14, respectively. (**E**) Representative images of the non-perfusion area on days one and eight. Scale bar shows 500 µm. (**F**–**G**) Quantitative data of the ratio of the non-perfusion area. ASP4058 treatment in the early phase (**F**) and late phase (**G**) inhibited the formation of the non-perfusion region. Data are presented as the mean ± SEM (n = 7–10). ^#^; *p* < 0.05, ^##^; *p* < 0.01 vs. vehicle-administered group (Welch’s *t*-test).

We previously reported establishing a murine RVO model^17^. To determine the effect of ASP4058 on the RVO model, we determined the thickness of INL and the area of the non-perfused region. The oral administration of ASP4058 (0.01–3 mg/kg) suppressed the retinal edema dose-dependently (Fig. 5A–D), and 0.3 mg/kg ASP4058 12 h before and just after laser irradiation decreased the ratio of the non-perfused region at one and seven days after laser irradiation (Fig. 5E–F). Treatment with 0.3 mg/kg ASP4058 in the late phase also suppressed the formation of the non-perfused region (Fig. 5E, G).

### Effect of ASP4058 on VEGF production

**Figure 6:**
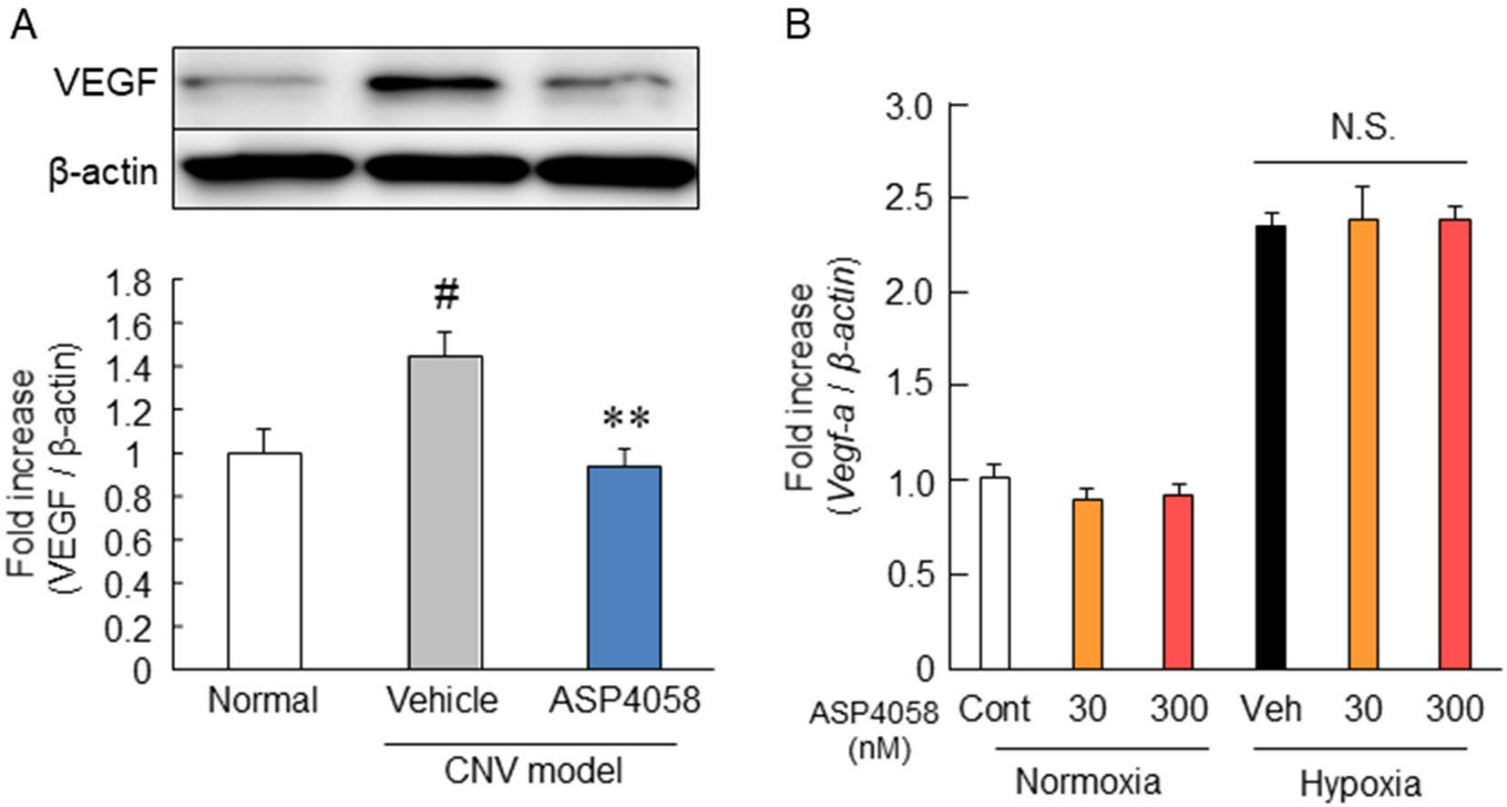
Effects of ASP4058 on VEGF production in in vivo and in vitro models. ASP4058 reduced the VEGF expression level in the CNV model mice. (**A**) Western immunoblotting analysis of VEGF and β-actin in the RPE-choroid complex five days after the laser irradiation. ASP4058 was orally administered once a day for six days from one day before laser irradiation. Data are presented as the mean ± SEM (n = 6–8). ^**^; *p* < 0.01 vs. vehicle-administered group, ^#^; *p* < 0.05 vs. normal group (Tukey’s test). (**B**) The presence or absence of the inhibitory effect of ASP4058 on VEGF mRNA expression was examined using RT-PCR. Pre-treatment of ASP4058 (final concentration: 30 or 300 nM) was started 1 h before hypoxia induction of treatment. Data are shown as the mean ± SEM (n = 6). N.S.; *p* > 0.05 vs. vehicle-treated group (Tukey’s test).

To reveal the mechanism that ASP4058 alleviated the chorioretinal pathologies, we performed a western blot analysis using a CNV model. We found that CNV induction increased the VEGF expression in the RPE-choroid complex, while ASP4058 reduced that compared to the vehicle-treated groups (Fig. 6A). Then, in vitro experiment by using ARPE-19 cells was conducted to determine whether ASP4058 affects *VEGF mRNA* production. As a result, ASP4058 did not suppress hypoxia-induced increase in *VEGF mRNA* expression (Fig. 6B). These results imply that ASP4058 does not directly inhibit VEGF production via S1P signaling.

## Discussion

Endothelial S1P has been reported to contribute to normalizing blood vessels^18^. As it has a different mechanism of action from anti-VEGF drugs, a new treatment for intraocular vascular lesions is expected. However, S1P3 signaling is associated with serious side effects^12,13^, making it difficult to develop an active compound against S1P3. We have already succeeded in synthesizing the S1P 1/5 selective agonist ASP4058, which has extremely weak activity against S1P3^14^. The current study investigated the efficacy of ASP4058 against intraocular angiogenesis and vascular permeability and examined whether it could be a candidate drug.

S1P1 was expressed in HRMECs as in other endothelial cells^19^. ASP4058 is presumed to have a high affinity for HRMECs. ASP4058 showed a concentration-dependent inhibitory effect on VEGF-induced cell proliferation and was not cytotoxic at the highest concentration of 300 nM. It also suppressed the disruption of intercellular junction protein, significantly inhibiting the increased permeability in a concentration-dependent manner. In an ex vivo rat aortic ring explant assay, S1P1 antagonist was reported to promote vigorous angiogenesis, while S1P1 agonist inhibited angiogenesis^11^. S1P1 agonist also suppressed the decreased expression of VE-cadherin by VEGF, while the S1P1 antagonist downregulated VE-cadherin^11^. The current results are in agreement with these previous reports, indicating that S1P signaling directly induces vascular stability in endothelial cells. Interestingly, ASP4058 inhibited phosphorylation of p38 and Src by VEGF, indicating that S1P agonist may inhibit intracellular VEGF signaling.

S1P1 expression in the RPE-choroid complex, including the lesion area of the CNV model, decreased at the protein level, although there was a transient point of increase in mRNA. The decreased expression of S1P1, which contributes to vascular homeostasis, is hypothesized to cause abnormal angiogenesis and disruption of barrier function in abnormal vessels. This is consistent with the typical phenotype in CNV models in which abnormal angiogenesis is accompanied by increased vascular permeability. S1P1 in the CNV model is expressed in endothelial and Müller glial cells, which are localized in the CNV and its surroundings^20,21^. FTY720 was phosphorylated in vivo and has been demonstrated to exert anti-inflammatory effects on astrocytes by regulating the release of inflammatory cytokines^22^. Therefore, S1P1 in glial cells that accumulate at CNV sites may be aimed at activating S1P signals to suppress the secretion of inflammatory cytokines, suggesting a type of homeostasis that inhibits the pathologic progression. Nonetheless, the role of S1P signaling in Müller glial cells and astrocytes in CNV pathogenesis remains unclear.

Oral administration of ASP4058 had a remarkable inhibitory effect on vascular hyperpermeability and choroidal neovascularization in the CNV model mice that mimicked exudative AMD^23^. Recently, it has been reported that siponimod, showing specificity for S1P1/5, could prevent the progression of suture-induced corneal neovascularization in rabbits. Modulation of S1PR signaling with S1P antibodies or FTY720 has already been reported to be effective in suppressing CNV^24–26^ and vascular inflammation^27^, and the present experimental results support these previous reports. The efficacy of FTY720 was verified at 0.3 mg/kg orally, the same dose that was shown to be effective in ASP4058; nonetheless, no significant effect was observed (Supplementary Fig. 4). However, a previous study reported the effect of FTY720 in the CNV model mice^26^. The reason for the difference between the results may be because the previous report may have shown a stronger protective effect with earlier administration than the present experiment. The efficacy of oral administration of ASP4058 against intraretinal edema was tested using RVO model mice. The minimum and maximum effective concentrations of ASP4058 for oral administration to RVO model mice were found to be approximately 0.01 and 1 mg/kg, respectively. ASP4058 has shown an inhibitory effect on retinal vascular permeability with a low dose of 0.01 mg/kg and is expected to be a drug that supports retinal vascular homeostasis. ASP4058 was effective from 1 nM in an in vitro assay using HRMECs, suggesting that ASP4058 is a compound that can inhibit the expansion of the intercellular spaces in retinal vascular endothelial cells from extremely low doses. Oral administration of ASP4058 suppressed the development of ischemic areas of retinal vessels in RVO model mice. We have previously shown that the administration of anti-VEGF-neutralizing antibodies further exacerbates retinal ischemia in RVO model mice^28^. Clinically, development of the ischemic area of the RVO is serious and can lead to neovascular glaucoma and irreversible vision loss. Therefore, it is medically significant to advocate the activation of S1P1 in the treatment of RVO, in which anti-VEGF therapy is the mainstay.

It is possible that continuous activation of S1P1 is essential for cortical actin formation in endothelial cells, which may be related to the maintenance of tight-junction protein complexes^29^. Another possibility is that S1P1 regulates the phosphorylation of tight-junction proteins^30^, which may enhance intercellular binding. Nevertheless, the detailed molecular mechanisms by which S1P1 signaling maintains endothelial cell intercellular homeostasis are unknown. Oral administration of ASP4058 inhibited the VEGF production in the RPE-choroid complexes of CNV model mice. Therefore, ASP4058 may be useful for normalizing choroidal vascular hyperpermeability and neovascularization. As activation of S1P suppresses HIF-1 signaling^31^, it is reasonable that ASP4058 administration leads to improvement of ischemia and suppression of VEGF expression in the eye. We used human retinal pigment epithelial cells, which are known to routinely produce VEGF in the eye. Contrary to expectations, ASP4058 did not suppress hypoxia-induced increase in VEGF mRNA expression. The suppression of VEGF production by ASP4058 in mouse choroidal tissues may be due to suppression of pathological progression by activation of S1P signaling. Although it is unclear whether Müller glia S1P1 is a target for reducing VEGF production in the choroidal complex, the strong expression of S1P1 in Müller glia accumulating at the lesion site suggests that the administered ASP4058 has some effect on Müller glial cells through S1P1. ASP4058 has several strengths; it differentiates itself from existing anti-VEGF therapies in terms of mechanism, it can be administered orally, and there is less concern about serious side effects such as bradycardia because it has no affinity for S1P3.

This study has several limitations. First, it was not possible to calculate the extent to which ASP4058 migrated intraocularly by oral administration. After 14 days of repeated oral administration of ASP4058 (0.1 mg/kg), the maximum plasma concentration of ASP4058 was 16.4 ± 0.463 ng/ml in Lewis rats^14^. ASP4058 was effective from 1 nM in vitro. Oral administration of ASP4058 at doses of 0.01 mg/kg has shown inhibitory effects against intraretinal edema and retinal vascular permeability. Even if we took species difference into account, we speculate that the concentration of ASP4058 shown to be effective in vivo in this study had reached or exceeded the concentration that would be effective in vitro. Second, as ASP4058 activates both S1P1 and S1P5, it is unknown whether the present effect is due to S1P1, S1P5, or both.

In summary, our data indicate that the oral administration of the S1P1/5 agonist ASP4058 could be a potential drug for ocular diseases with vascular abnormalities such as AMD and RVO. Because existing anti-VEGF therapies are administered intravitreally, there is a need for other drugs that could help improve adherence.

## Supplementary Information

Supplementary Figures.

## Data Availability

Data supporting the findings of this manuscript are available from the corresponding author upon reasonable request.
