# Correction: Sphingosine-1-phosphate receptor 1/5 selective agonist alleviates ocular vascular pathologies

**DOI:** 10.1038/s41598-026-57359-8

**Published:** 2026-07-02

**Authors:** Shinsuke Nakamura, Rie Yamamoto, Takaya Matsuda, Hiroto Yasuda, Anri Nishinaka, Kei Takahashi, Yuki Inoue, Sadao Kuromitsu, Masamitsu Shimazawa, Masahide Goto, Shuh Narumiya, Hideaki Hara

**Affiliations:** 1 Molecular Pharmacology, Department of Biofunctional Evaluation, Gifu Pharmaceutical University, 1‑25‑4 Daigaku‑nishi, Gifu, 501‑1196 Japan; 2 Discovery Accelerator, Astellas Pharma Inc., Tsukuba, Japan; 3 Alliance Laboratory for Advanced Medical Research, Kyoto University Graduate School of Medicine, Kyoto, Japan; 4 Pharmaceutical Research and Technology Labs, Astellas Pharma Inc., Yaizu, Japan; 5 Astellas Institute for Regenerative Medicine, Marlborough, MA USA; 6 Department of Drug Discovery Medicine, Kyoto University Graduate School of Medicine, Kyoto, Japan

Correction to: *Scientific Reports* 10.1038/s41598-024-60540-6, published online 27 April 2024

The original version of this Article contained an error in Figure 5A, where the image for ASP4058, 3mg/kg (far right, bottom row) was derived from the same source data as the image for ASP4058, 1mg/kg. The error arose during figure assembly and is now updated with the correct source data. The original Figure 5 and accompanying legend appear below.

**Fig. 5:**
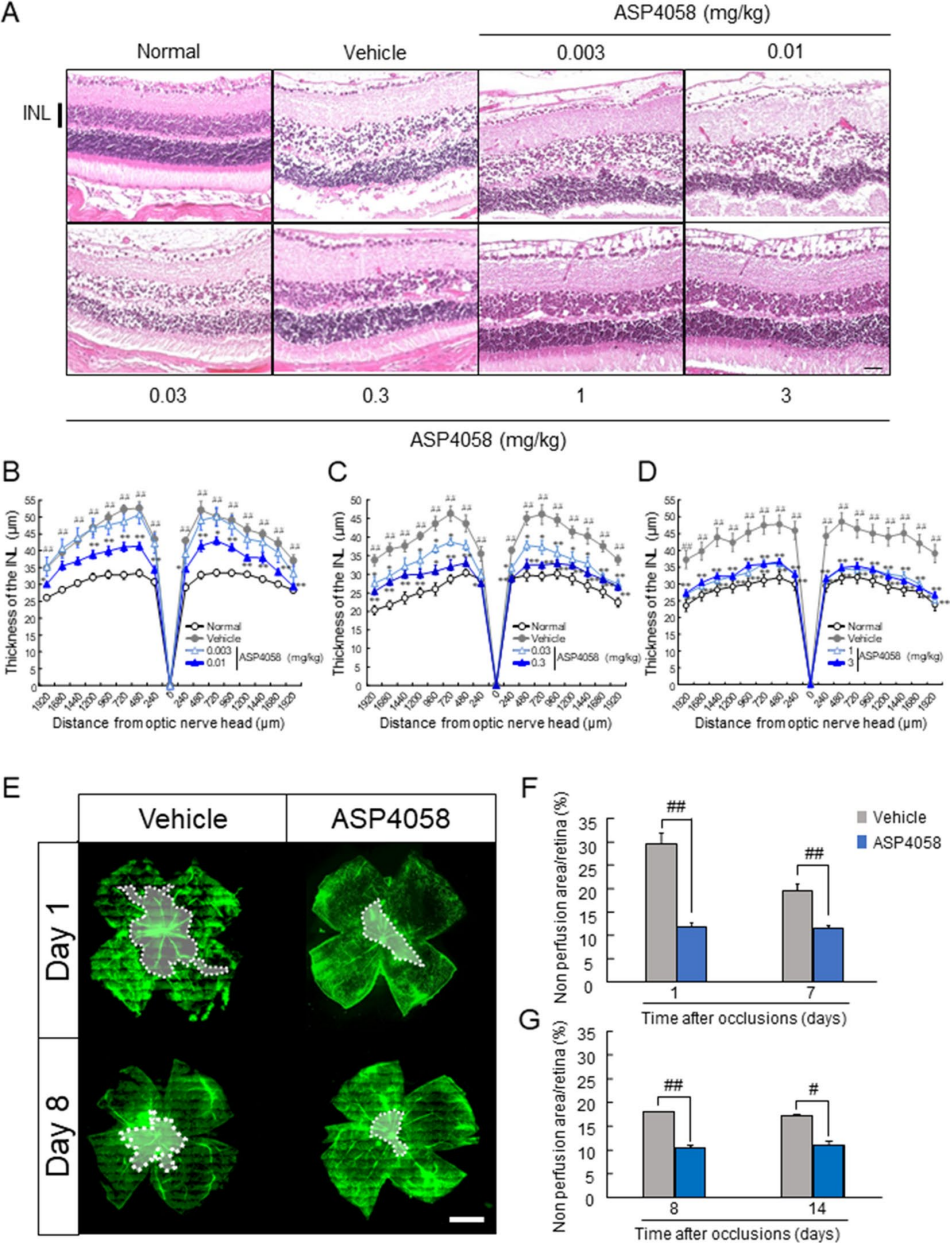
Effect of ASP4058 on the formation of retina edema and non-perfused region in the RVO model mice. ASP4058 suppressed the formation of retinal edema and non-perfusion area in the RVO model. (**A**) Photomicrographs of representative H&E-stained retinal sections of normal, vehicle, and ASP4058 groups at 0.003, 0.01, 0.03, 0.3, 1, and 3 mg/kg orally administered just after laser irradiation. Images were taken at 500 µm from the optic nerve head. Scale bar indicates 50 µm. (**B**–**D**) Quantitative data of the thickness of the INL. Each graph contains 0.003 and 0.01 (**B**), 0.03 and 0.3 (**C**), and 1 and 3 mg/kg (**D**) ASP4058. Data are presented as the mean ± SEM (n = 10). ^*^; *p* < 0.05, ^**^; *p* < 0.01 vs. vehicle-treated group (Dunnett’s T3 test), ^##^; *p* < 0.01 vs. normal group (Welch’s *t*-test). (**E**–**G**) The effect of ASP4058 at 0.3 mg/kg on the formation of a non-perfusion area. ASP4058 was orally administered 12 h before and just after laser irradiation (early phase) or twice seven days after occlusion (late phase). To examine the effect of ASP4058 treatment in the early and late phase, the retinas were collected at days one or seven, and eight or 14, respectively. (**E**) Representative images of the non-perfusion area on days one and eight. Scale bar shows 500 µm. (**F**–**G**) Quantitative data of the ratio of the non-perfusion area. ASP4058 treatment in the early phase (**F**) and late phase (**G**) inhibited the formation of the non-perfusion region. Data are presented as the mean ± SEM (n = 7–10). ^#^; *p* < 0.05, ^##^; *p* < 0.01 vs. vehicle-administered group (Welch’s *t*-test).

The original Article has been corrected.

