# Supplementary material for: Sphingosine-1-phosphate receptor 1/5 selective agonist alleviates ocular vascular pathologies
Source: Sci Rep. 2024 Apr 27;14:9700. doi: 10.1038/s41598-024-60540-6 (PMC11055896; doi:10.1038/s41598-024-60540-6)
Supplement: Supplementary file 1 — Supplementary Figures. [file 41598_2024_60540_MOESM1_ESM.pdf]

Supplementary information

Sphingosine-1-phosphate receptor 1/5 selective agonist alleviates ocular vascular pathologies

Shinsuke Nakamura,[1] Rie Yamamoto,[2,3] Takaya Matsuda,[4] Hiroto Yasuda,[1] Anri Nishinaka,[1] Kei Takahashi,[1] Yuki Inoue,[5] Sadao Kuromitsu,[2,3] Masamitsu Shimazawa,[1] Masahide Goto,[5] Shuh Narumiya,[6] Hideaki Hara[1,*]

Affiliations:

[1]Molecular Pharmacology, Department of Biofunctional Evaluation, Gifu Pharmaceutical University, Gifu, Japan

[2]Discovery Accelerator, Astellas Pharma Inc., Tsukuba, Japan

[3]Alliance Laboratory for Advanced Medical Research, Kyoto University Graduate School of Medicine, Kyoto, Japan

[4]Pharmaceutical Research and Technology Labs, Astellas Pharma Inc., Yaizu, Japan

[5]Astellas Institute for Regenerative Medicine, MA, USA

[6]Department of Drug Discovery Medicine, Kyoto University Graduate School of Medicine, Kyoto, Japan

***Correspondence and reprint requests to:**

President Hideaki Hara, R.Ph., Ph.D.

Gifu Pharmaceutical University, 1-25-4 Daigaku-nishi, Gifu 501-1196, Japan.

Phone/Fax: +81-58-230-8150, E-mail: hidehara@gifu-pu.ac.jp

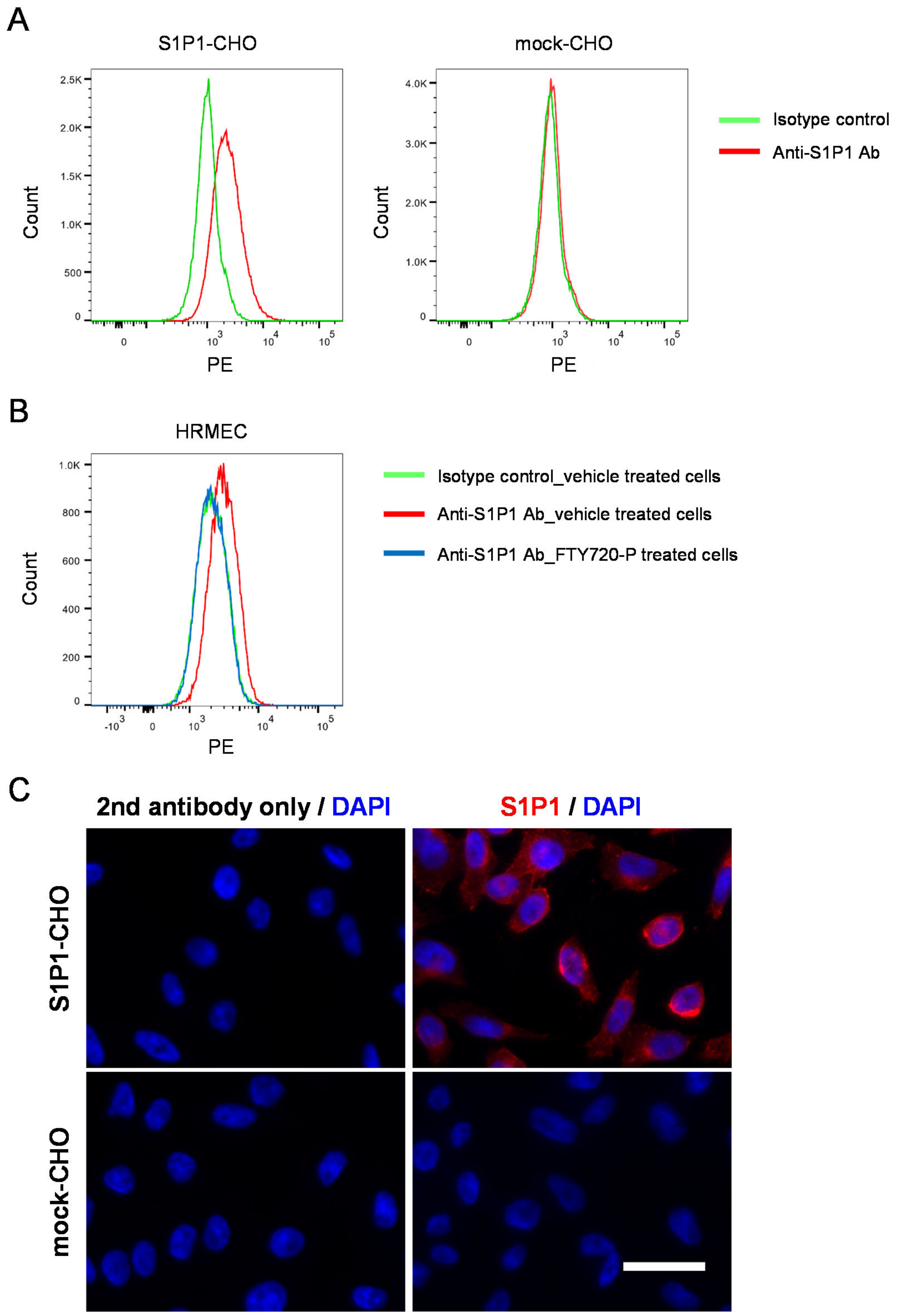

Supplementary figure 1. The specificity of the anti-S1P1 antibody.

(A) The specificity of the anti-S1P1 antibody for FACS analysis was investigated using CHO cells with forced expression of S1P1- and mock-CHO cells. (B) FTY720-P attenuated S1P1 signaling on HRMECs. (C) Representative immunofluorescence image of S1P1 receptor expression of S1P1- and mock-CHO cells. Scale bar = 30 μm.

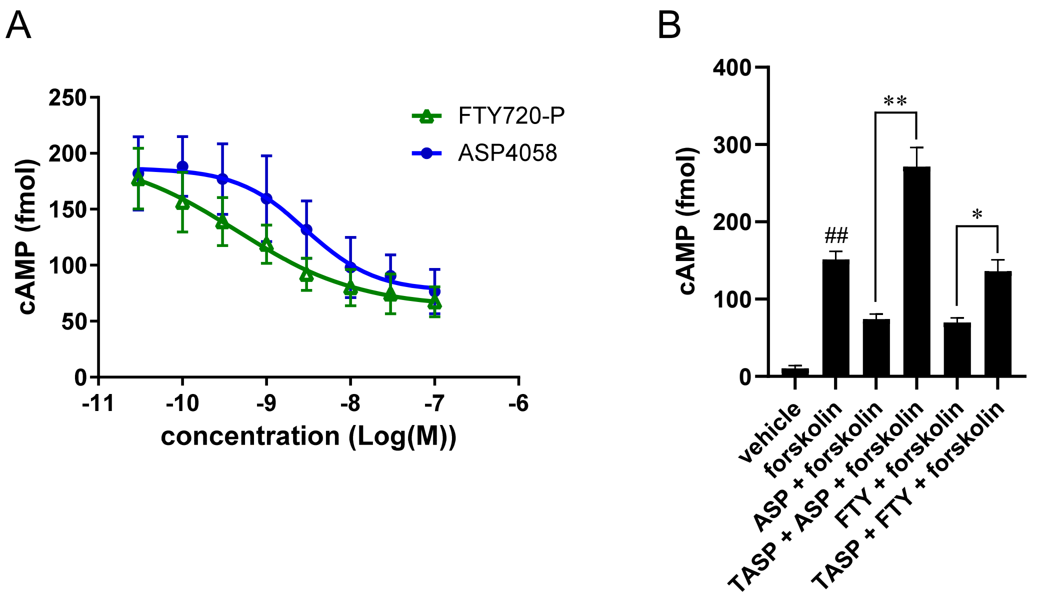

Supplementary figure 2. S1P1-mediated inhibition of forskolin-stimulated cAMP accumulation. (A) Both ASP4058 and FTY720-P decreased cAMP in a concentration-dependent manner. (B) Both FTY720-P and ASP4058 reduced forskolin-induced cAMP accumulation. TASP0277308, a highly selective S1P1 antagonist, counteracted the effects of these compounds. Three independent experiments were performed in triplicate. $^{\#\#}$; $p<0.01$ vs. Vehicle-treated group, *; $p<0.05$ vs. FTY720-P and forskolin-treated group, **; $p<0.01$ vs. ASP4058 and forskolin-treated group (Tukey's test).

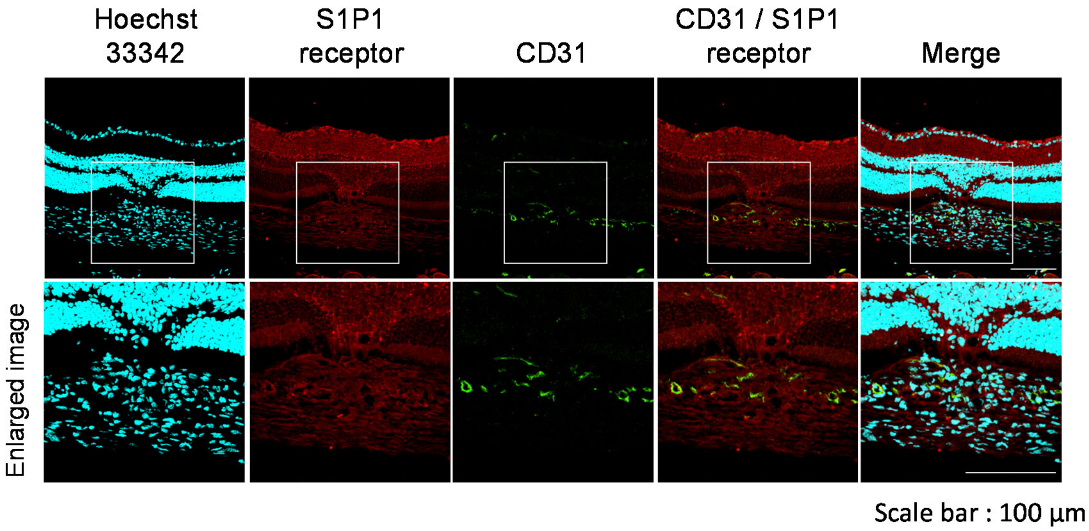

Supplementary figure 3. Localization of S1P1 receptor in and around CNV sites.

Representative immunofluorescence image of S1P1 receptor expression of choroidal neovascularization. Immunohistochemistry of Hoechst 33342 (cyan), S1P1 (red), CD31 (green). Scale bar shows 100 μm.

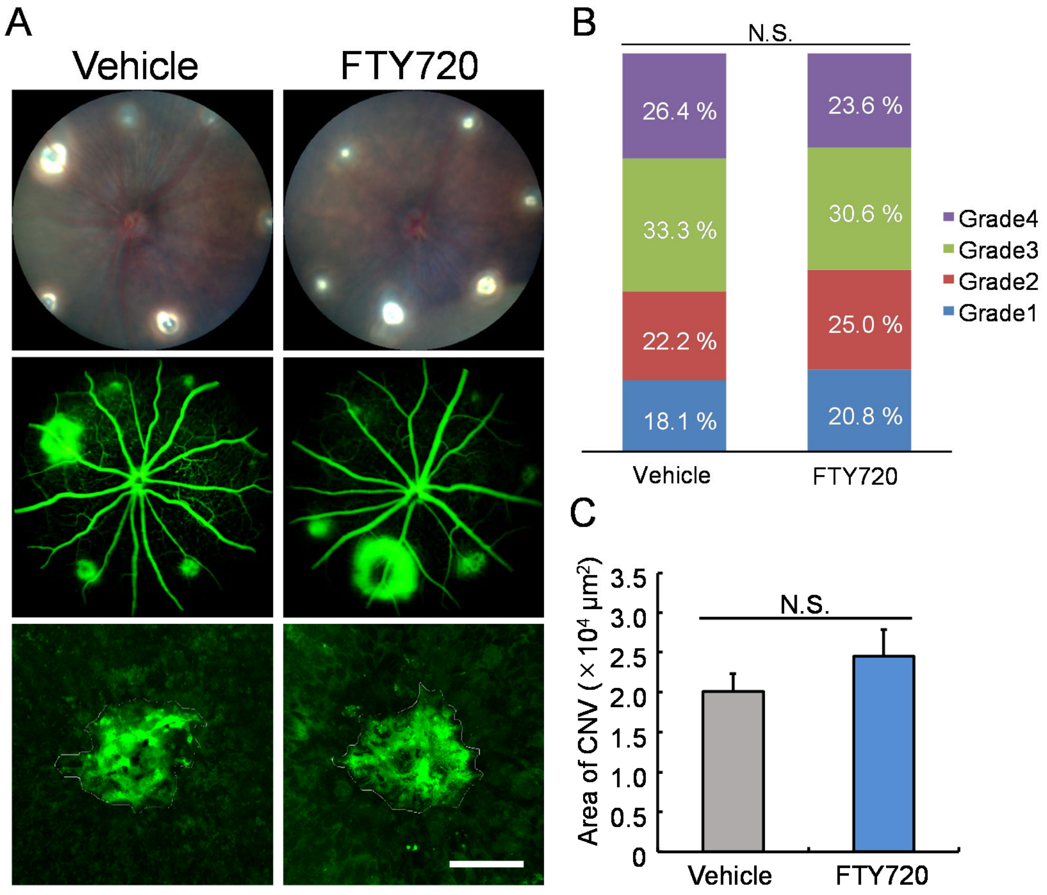

Supplementary figure 4. Anti-angiogenic effects of FTY720 on the laser-induced CNV model.

FTY720 did not show the anti-angiogenic effects in the laser-induced CNV model. (A-C) The murine laser-induced CNV model mice were orally administered FTY720 at 0.3 mg/kg once a day. (A) Representative fundus images just after laser irradiation (day zero) and after fluorescein injection (day 14) with grades (1-4) and CNV lesion visualized by FITC-dextran. (B) Quantitative data of vascular leakages (grade 1-4) of each spot. (C) Quantification of the mean size of CNVs. The scale bar shows 40 μm. Data are presented as the mean ± SEM (n= 12).

Figure 2E: Full scans of immunoblots.

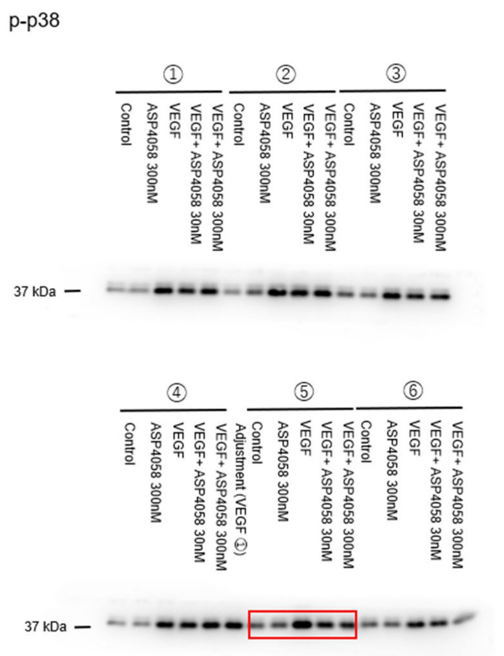

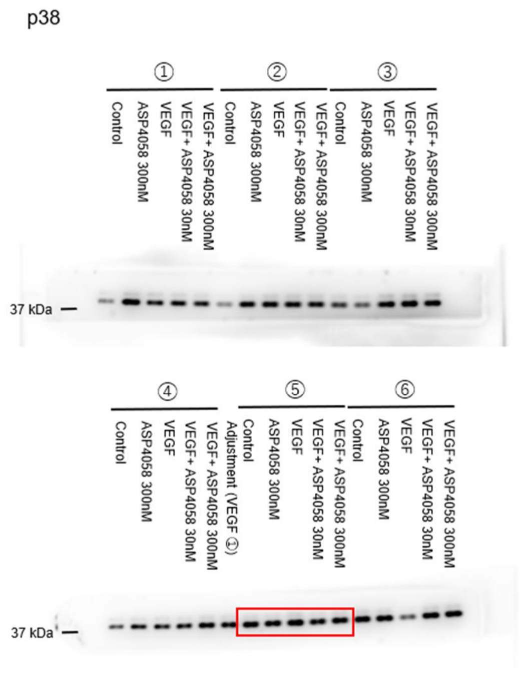

Contrast-modified Images

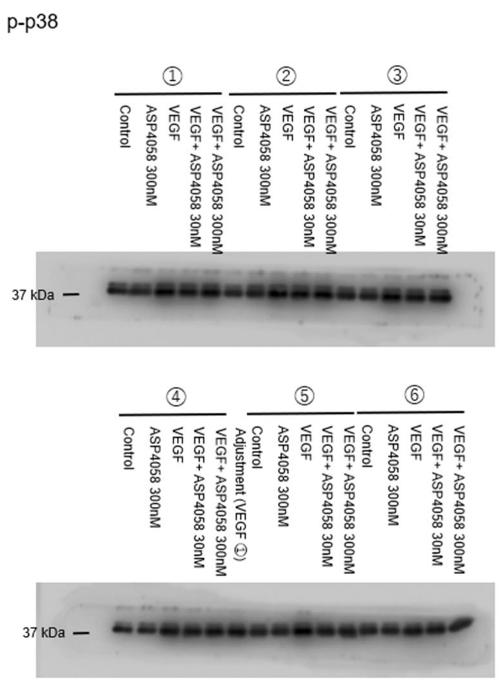

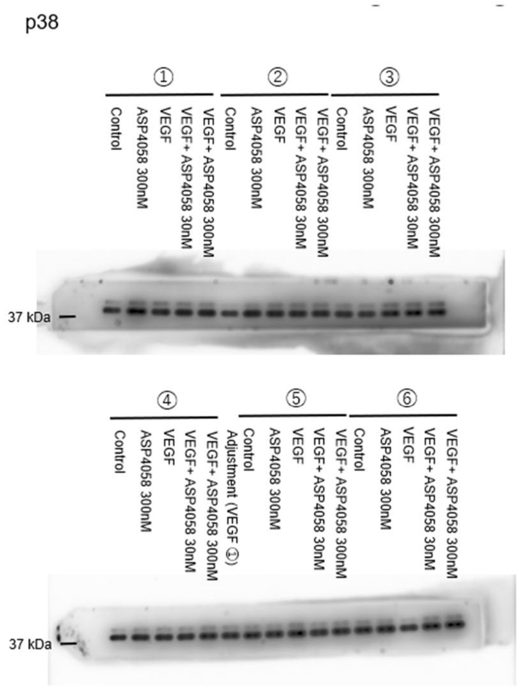

Figure 2E: Full scans of immunoblots.

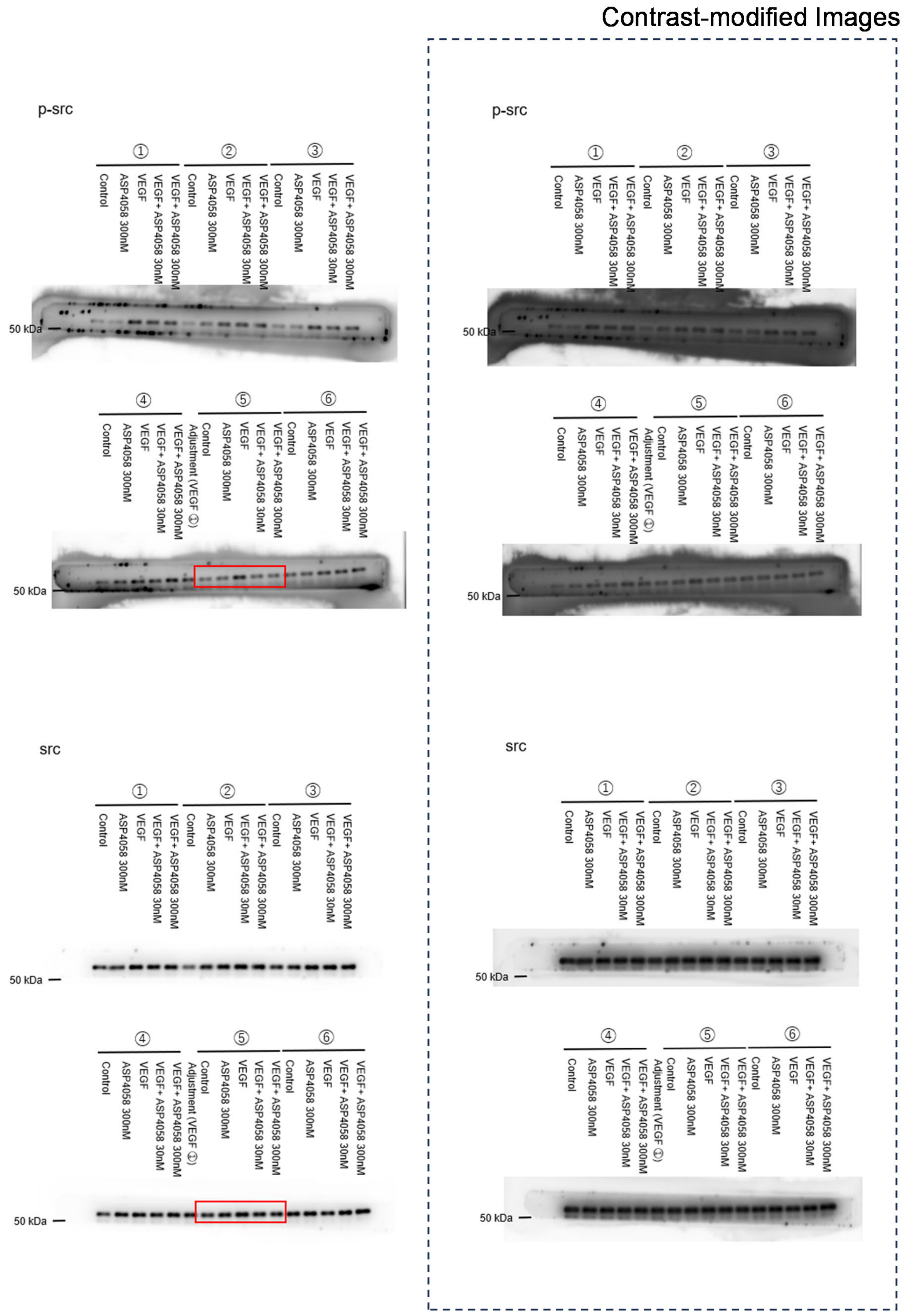

Figure 2E: Full scans of immunoblots.

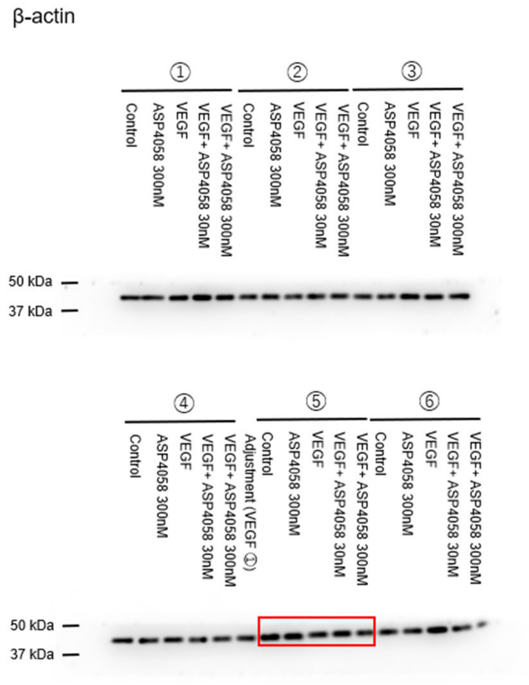

Contrast-modified Images

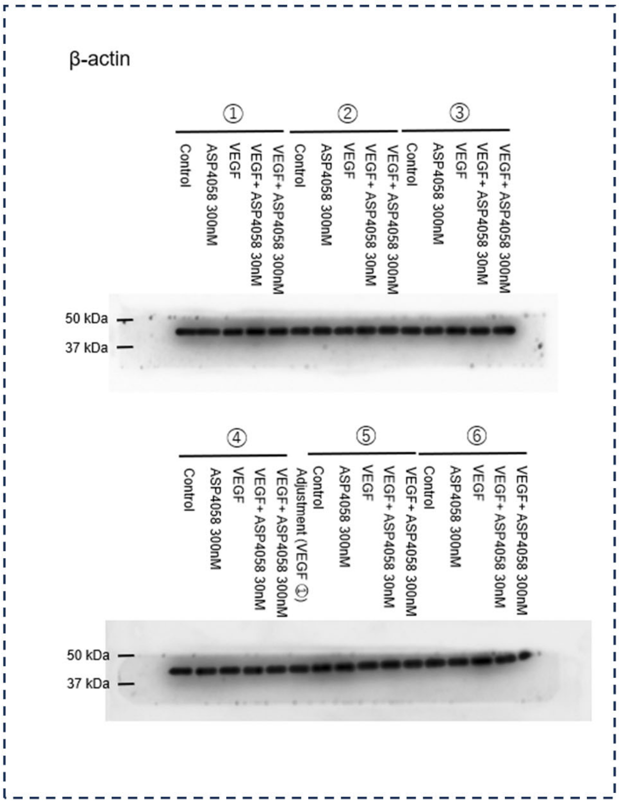

Figure 3B: Full scans of immunoblots.

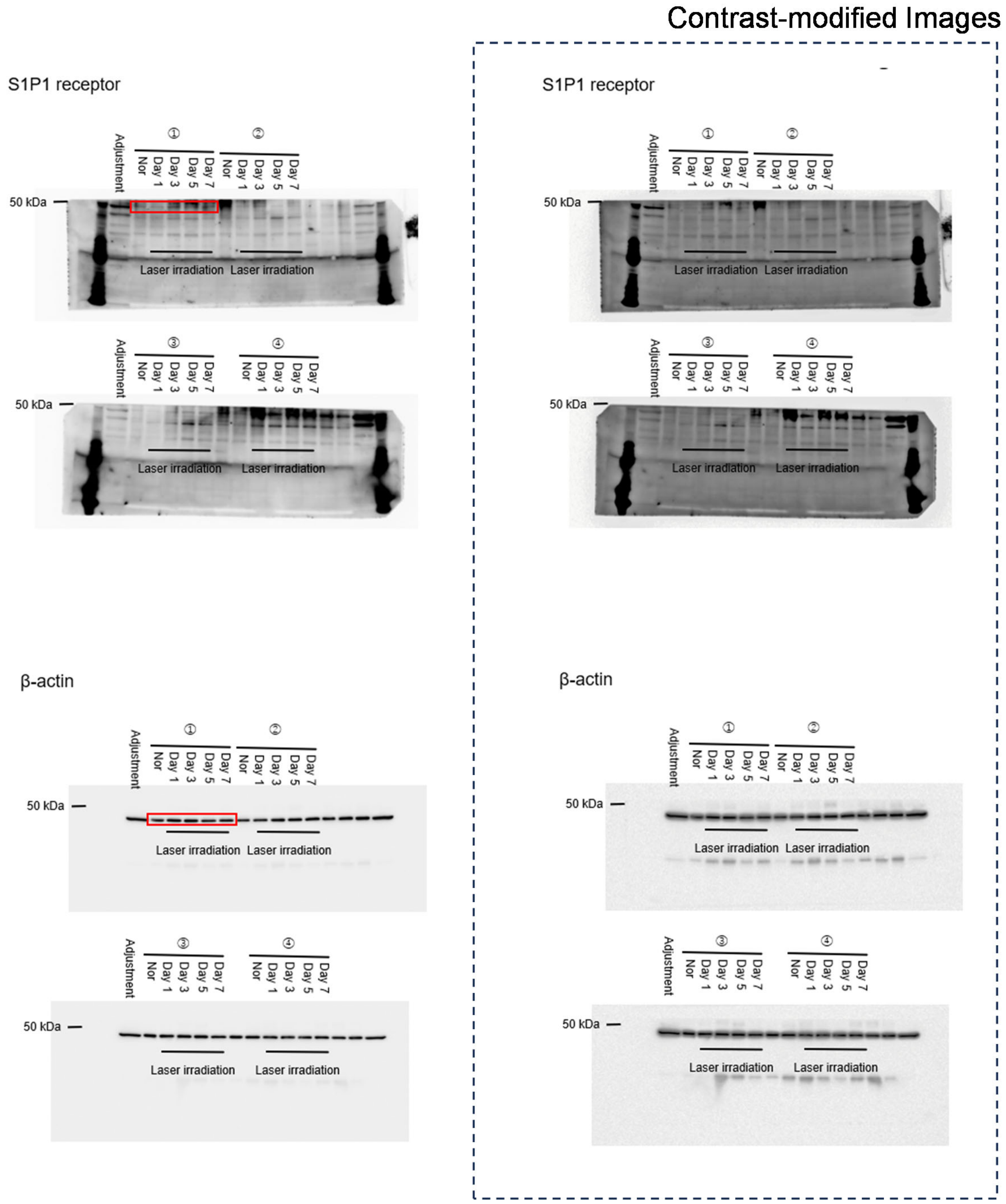

Figure 6: Full scans of immunoblots.

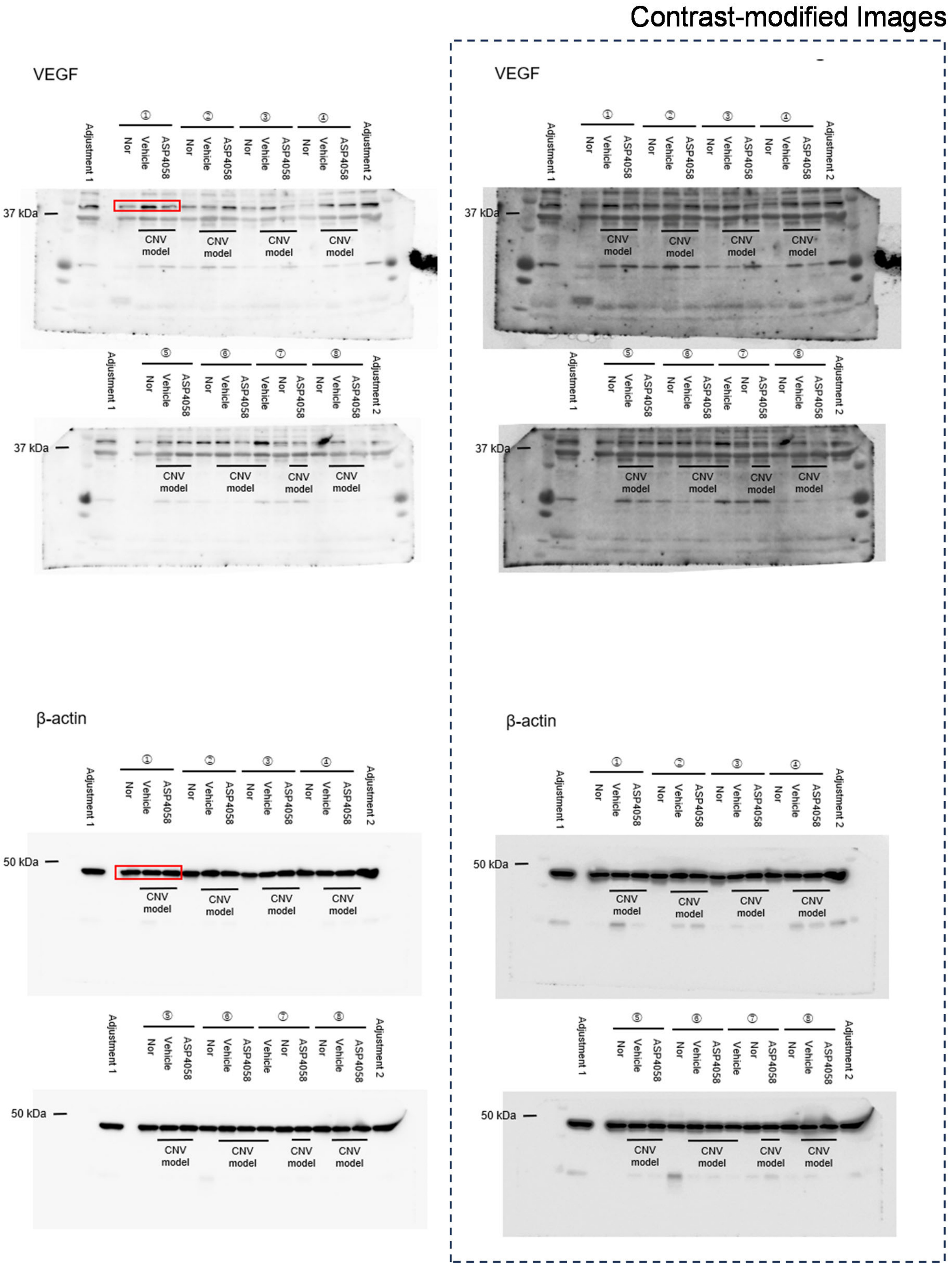

Supplementary Figure 5. Full scans of immunoblots.

The boxed regions are presented in the indicated figures. The original images (left) and the images with the contrast modified (right). The images on the right are modified for all proteins to make the full membrane clearer.
